# Robot‐Assisted, Conventional Fluoroscopy (C‐Arm), O‐Arm Navigation, and Freehand Pedicle Screw Fixation in Thoracolumbar Spine Fracture Surgery: A Network Meta‐Analysis

**DOI:** 10.1111/os.70189

**Published:** 2025-10-11

**Authors:** Yankun Zhu, Shuaiqi Zhu, Yanan Li, Kun Wang

**Affiliations:** ^1^ Department of Orthopaedics Union Hospital, Tongji Medical College, Huazhong University of Science and Technology Wuhan China; ^2^ Department of Statistics Honours College, Capital Normal University Beijing China

**Keywords:** fluoroscopy, network meta‐analysis, O‐arm, pedicle screw fixation, robot, thoracolumbar spine fracture

## Abstract

Thoracolumbar fractures are a prevalent clinical disease, with several surgical techniques, including traditional freehand pedicle screw fixation (TFPSF), conventional fluoroscopy (C‐arm) percutaneous pedicle screw fixation (CPPSF), O‐arm‐assisted percutaneous pedicle screw fixation (OPPSF), and robot‐assisted percutaneous pedicle screw fixation (RPPSF), being currently applied. However, a comprehensive comparison of their relative efficacy across multiple perioperative and functional outcomes is lacking, leading to uncertainty in optimal technique selection. This network meta‐analysis (NMA) evaluates and compares the clinical efficacy of these four surgical techniques to identify the most effective intervention and guide clinical decision‐making. Researchers independently searched PubMed, Embase, the Cochrane Central Register of Controlled Trials, and Web of Science for studies published before September 20, 2024. Studies were selected based on stringent eligibility criteria. Randomized controlled trials (RCTs) were assessed using RoB 2.0, while cohort studies were evaluated with the Newcastle–Ottawa Scale (NOS). After data extraction, Bayesian network analysis was executed using R 4.2.2 and Stata 16.0. Nineteen studies were included, encompassing 1344 patients with thoracolumbar fractures. For screw accuracy, OPPSF ranked highest (SUCRA = 92.7%), significantly outperforming TFPSF (RR 1.12; 95% credible intervals [CrI] [1.04, 1.23]) and CPPSF (RR 1.12; 95% CrI [1.04, 1.22]), with RPPSF also surpassing both. OPPSF showed superior intraoperative blood loss reduction (SUCRA = 79.8%) while TFPSF had significantly more bleeding than others. For hospitalization, RPPSF ranked highest (SUCRA = 65.0%) but CPPSF significantly shortened stays versus TFPSF (MD −2.24; 95% CrI [−4.48, −0.03]). CPPSF also showed better pain control (SUCRA = 77.9%) with significantly lower VAS scores versus TFPSF (MD −1.02; 95% CrI [−1.71, −0.37]). RPPSF demonstrated the lowest complication risk (SUCRA = 94.9%), with both CPPSF and RPPSF showing significant reductions versus TFPSF. Additionally, although CPPSF ranked first in SUCRA for both operative time (SUCRA = 81.6%) and Cobb angle (SUCRA = 72.4%), the pairwise comparisons did not demonstrate statistical significance, necessitating cautious interpretation. In summary, OPPSF tends to demonstrate superior precision and blood loss control, CPPSF may optimize rehabilitation efficiency, while RPPSF appears to be the safest technique. Technique selection should balance clinical outcomes, economic feasibility, and patient‐specific priorities.

## Graphical abstract

Bayesian network meta‐analysis of four surgical techniques for thoracolumbar fractures revealed that OPPSF tends to demonstrate superior precision and blood loss control, CPPSF may optimize rehabilitation efficiency, while RPPSF appears to be the safest technique.

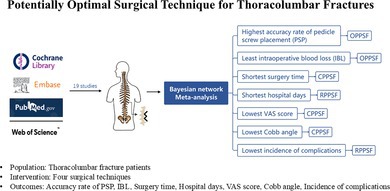

## 1 Introduction

The prevalence of spinal fractures has been continuously growing owing to the speedy development of urban construction and the rise in traffic accidents [1, 2]. Among these, thoracolumbar fractures, predominantly resulting from high‐energy trauma such as motor vehicle collisions and falls [2, 3, 4, 5, 6, 7], are the most common type [8] and are often observed in adult males [1, 2, 3, 4]. Approximately 60%–70% occur at the thoracolumbar junction (T12‐L2), a biomechanically vulnerable transition zone [3, 5, 7, 9, 10, 11, 12, 13]. These injuries compromise spinal integrity and stability [13], typically presenting with localized pain, restricted mobility, neurological deficits, and potential bowel or bladder dysfunction [7]. Common treatment modalities for such fractures include conservative management and surgical intervention [7, 14], among which posterior fixation using pedicle screws is the most frequently employed surgical technique [10]. At present, multiple surgical approaches have been developed [11, 12, 15], but studies reveal significant heterogeneity in their perioperative outcomes, including operative time, blood loss, and hospitalization duration [16, 17, 18, 19, 20].

For this reason, several surgical techniques were selected for the study, which are among the most common, representative, and promising in clinical practice. These techniques include traditional freehand pedicle screw fixation (TFPSF) and percutaneous pedicle screw fixation (PPSF). The latter, PPSF, can be further categorized into conventional fluoroscopy (C‐arm) percutaneous pedicle screw fixation (CPPSF), O‐arm‐assisted percutaneous pedicle screw fixation (OPPSF), and robot‐assisted percutaneous pedicle screw fixation (RPPSF). Previous studies have demonstrated that these surgical techniques have their respective advantages and disadvantages with regard to hospitalization length, IBL, operative time, and intraoperative radiation exposure time [17, 18, 19, 20]. Among them, the studies conducted by Tu et al. and Bronsard et al. demonstrated that the surgical duration and hospitalization period for TFPSF were significantly longer, the IBL was greater, and the incidences of postoperative pain and complications were markedly higher compared to percutaneous minimally invasive pedicle screw fixation (PMIPSF); however, the duration of intraoperative radiation exposure was notably lower in the TFPSF group [21, 22]. For PMIPSF, CPPSF enables low VAS scores and good pain control. However, it is related to prolonged operative time and increased duration of radiation exposure [22]. In comparison, OPPSF can shorten operative time, increase screw placement accuracy, and reduce radiation exposure [23]. Lin et al.'s research indicates that the accuracy of screw placement in RPPSF is superior to that of CPPSF. Significant differences were not noted between CPPSF and RPPSF concerning IBL, duration of hospitalization, or complication rates. However, the surgical costs and incision length were higher in RPPSF compared to CPPSF [24]. Nevertheless, a significant evidence gap persists. The absence of a unified consensus and the reliance on pairwise comparisons, which cannot provide a hierarchical ranking of all techniques, create considerable uncertainty for surgeons in selecting the optimal approach to meet the diverse needs and specific conditions of individual patients in clinical practice. Crucially, while pairwise meta‐analyses have provided fragmented comparisons, there is a complete lack of a network meta‐analysis (NMA), which could simultaneously and comprehensively compare these four interventions across multiple critical outcomes. This evidence gap makes the results of some key comparisons, particularly those between the newer technologies like OPPSF and RPPSF, remain inconclusive, directly impeding optimal surgical decision‐making.

In response to these issues, this NMA was carried out. NMA integrates both direct (direct comparisons between different interventions) and indirect evidence (indirect comparisons through common comparators), providing a comprehensive framework for comparing multiple interventions. This characteristic enables NMA to address the lack of direct comparison results between certain interventions, and it also offers probability rankings for various interventions based on different outcome measures. This study compares TFPSF, CPPSF, OPPSF, and RPPSF to determine the optimal intervention based on probability ranking, thus providing evidence to support clinical decision‐making and offering a theoretical foundation for future research.

## 2 Method

### 2.1 Design and Registration

The NMA was undertaken following the preferred reporting items for systematic reviews and meta‐analyses (PRISMA) [25] evaluation guidelines. Our study has been registered in the International Prospective Systematic Review Register (PROSPERO) with the identifier CRD42024592811.

### 2.2 Search Methods

Researchers independently conducted a literature search in PubMed, Embase, the Cochrane Central Register of Controlled Trials, and Web of Science for articles before September 20, 2024, without restrictions on document type, date/time, or publication status. Both Medical Subject Headings (MeSH) and free‐text keywords were used for the search, including all known spellings of terms such as “thoracolumbar fracture,” “pedicle screws,” “freehand,” “fluoroscopy,” “robot‐assisted surgery,” and “O‐Arm” (for the detailed search strategy, refer to the Data S1). A manual search of relevant literature was also performed to ensure no studies were missed.

### 2.3 Inclusion and Exclusion Criteria

The criteria for inclusion and exclusion were designed on the basis of the Participants, Intervention, Control, Outcome, and Study Design (PICOS).

Inclusion criteria included: (1) patients diagnosed with thoracolumbar vertebral fractures at any age; (2) patients in the intervention group underwent TFPSF, RPPSF, CPPSF, or OPPSF; (3) those in the control group received one of the foregoing surgical procedures; if both intervention and control groups had general adjunctive therapy, the adjunctive therapy should be identical; (4) the study reported at least one of these outcomes: accuracy rate of pedicle screw placement (PSP) based on the Gertzbein–Robbins scale [26] or other standardized grading systems, IBL, surgical duration, hospital days, Visual Analog Scale (VAS) score, Cobb angle, as well as the incidence of complications; (5) the study design type was required to be either a cohort study or a randomized controlled trial (RCT); (6) studies must be written in English.

Exclusion criteria included: (1) review articles, case reports, descriptive studies, opinion articles, conference papers, or abstracts; (2) studies with no full texts or those for which the full text could not be retrieved; (3) studies with erroneous or incomplete data that could not be pooled; (4) interventions involving other therapeutic modalities; (5) studies that did not report the aforementioned outcome measures or lacked clear statistical standards.

### 2.4 Study Selection

Following the pre‐established eligibility criteria, two researchers independently performed literature screening. Initially, all potentially relevant studies were uploaded to EndNote 21 for the removal of duplicates. Subsequently, titles and abstracts were screened to ostracize ineligible ones. The full texts of those left after the initial screening were retrieved and reviewed, and the studies were further assessed to determine eligible studies. Disagreement was addressed through consulting a third researcher for discussion or resolution.

### 2.5 Data Extraction

The standardized Excel spreadsheet created was utilized to extract the data as follows: (1) basic information like the first author's name, publication date, and study design type; (2) characteristics of study participants, including age, sex, nationality, sample size for each group, and average follow‐up duration; (3) intervention methods; (4) outcomes, including the accuracy rate of PSP, IBL, surgery duration, hospital stay, VAS score at the last follow‐up, Cobb angle at the last follow‐up, and incidence of complications. Additionally, only Grade A (no screw breach) and Grade B (< 2 mm breach) were considered as accurate placement according to the Gertzbein–Robbins scale [26]. Two researchers independently extracted data from the eligible studies based on the preprepared standardized spreadsheet. Subsequently, the two researchers cross‐checked their data, and in case of any discrepancies, they consulted a third party.

### 2.6 Quality Assessment

#### 2.6.1 Cohort Studies

Two independent researchers rated the quality of eligible studies via the Newcastle–Ottawa Scale (NOS) [27], which evaluates study quality across three domains with eight questions. Besides comparability, which is scored out of 2 points, the other seven questions are scored out of 1 point each. A total score of 7–9 was indicative of high‐quality studies, while 4–7 points indicated moderate quality. After completing the assessment, the two researchers cross‐checked their evaluation results. In case of disagreement, a third researcher assisted in resolving the discrepancies.

#### 2.6.2 RCTs

Two independent researchers evaluated the risk of bias in RCTs through the Cochrane Risk of Bias Tool, version 2 (RoB 2.0) [28]. Each study was rated in these domains: bias arising from the randomization process, bias owing to deviations from intended interventions, bias due to missing outcome data, bias in outcome measurement, and bias in reported results selection, including bias related to the registered protocol. A study was deemed to have an overall high risk of bias if at least one domain received a “high risk of bias” rating. Conversely, a study was considered to have an overall low risk of bias if every domain was evaluated as having a “low risk of bias.” After the assessments, the two researchers cross‐checked their evaluation results, and in cases of disagreement, a third researcher assisted in resolving the differences.

### 2.7 Data Synthesis and Statistical Analysis

#### 2.7.1 Primary Analysis

Statistical models based on the Bayesian framework were developed via JAGS (gemtc 0.8–2 and rjags 4–10 packages) within the R environment (version 4.2.2) (RStudio, Boston, MA, USA). The mean difference (MD) with a 95% credible interval (CrI) was derived for continuous data to assess the effect size. For categorical data, a pooled risk ratio (RR) with a 95% CrI was computed. Random‐effects models were employed for all NMAs due to the clinical heterogeneity of the included trials, which varied across countries, average ages, surgeons, surgical procedures, medical histories, and follow‐up durations. The surface under the cumulative rank curve (SUCRA) was used to examine the relative ranking of various surgical techniques for every outcome of interest [29]. A greater SUCRA value indicated a higher ranking of the intervention [29]. Additionally, the consistency and inconsistency models were compared via the deviance information criterion (DIC). If the DIC difference was below 5 points, the consistency model was appropriate [25]. To assess publication bias, comparison‐adjusted funnel plots were created. Network plots and comparison‐adjusted funnel plots for NMA were generated via Stata 16.0 (StataCorp, College Station, TX, USA).

#### 2.7.2 Sensitivity Analysis

To assess the robustness of the primary findings, two sensitivity analyses were performed to evaluate potential sources of bias. First, a dominant country analysis was conducted to investigate the potential influence of disparities in healthcare resources by restricting the analysis to studies originating from the country contributing more than 50% of the included studies. Second, a high‐quality study analysis was undertaken to reduce the confounding effects of lower‐quality studies; this analysis included only cohort studies with an NOS score ≥ 7 or RCTs evaluated as low risk of bias via RoB 2.0, with a minimum sample size of 20 participants per group and a follow‐up duration of at least 12 months. Both sensitivity analyses were carried out using the same Bayesian NMA framework as that employed in the primary analysis.

## 3 Results

### 3.1 Search Outcomes

**FIGURE 1:**
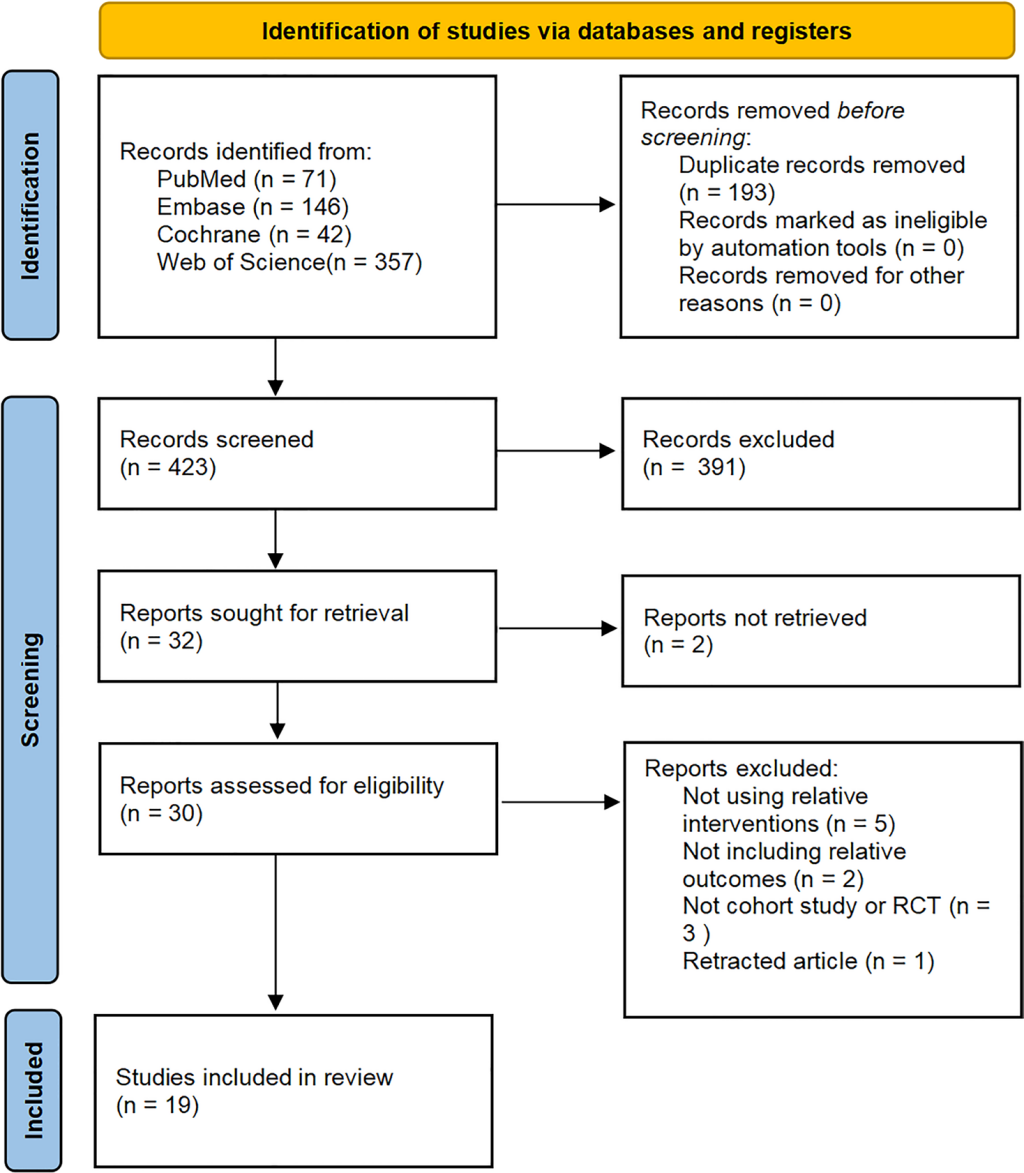
PRISMA flow diagram for search and selection of eligible studies in the NMA.

The detailed literature selection process is presented in the PRISMA flowchart in Figure 1. At first, 616 possibly associated studies were identified from the four databases. After 193 duplicates were ostracized, the titles and abstracts of the remaining studies were checked according to our eligibility criteria. Next, 391 studies were removed. The full text of the remaining 32 studies was further assessed. Two studies were excluded due to the unavailability of their full texts, while 11 studies were excluded for reasons including retraction, failure to utilize the relevant intervention, lack of reporting on relevant outcome measures, and noncompliance with the study design requirements (Figure 1). Ultimately, 19 studies were eligible for our NMA.

### 3.2 Characteristics of Included Studies

The characteristics and details of eligible studies are displayed in Table 1 [8, 18, 20, 21, 22, 24, 29, 30, 31, 32, 33, 34, 35, 36, 37, 38, 39, 40, 41]. Nineteen articles published between 2013 and 2023 were included [8, 18, 20, 21, 22, 24, 29, 30, 31, 32, 33, 34, 35, 36, 37, 38, 39, 40, 41], with 15 articles originating from China [8, 18, 20, 21, 24, 29, 32, 33, 34, 35, 36, 38, 39, 40, 41], one article from the United Kingdom [31], one article from France [22], one article from the United States [37] as well as one article from Saudi Arabia [30]. Overall, the sample sizes of these 19 studies were 37–130 individuals, with average ages ranging from 36.89 to 61.4 years, encompassing 1344 patients with thoracolumbar fractures. Among these, 17 studies included interventions involving CPPSF, with 565 patients in total [8, 21, 22, 24, 29, 32, 33, 34, 35, 36, 38, 39, 40, 41]. Thirteen studies involved TFPSF with 408 patients [8, 18, 20, 21, 22, 29, 30, 31, 32, 33, 34, 35, 41]. Six studies used RPPSF with 217 patients [20, 24, 29, 36, 37, 38]. Four studies involved OPPSF with 154 patients [18, 39, 40, 41]. These studies reported seven outcome measures: accuracy rate of PSP (*n* = 10) [18, 20, 24, 29, 36, 37, 38, 39, 40, 41], IBL (*n* = 14) [18, 20, 21, 22, 24, 30, 31, 32, 33, 34, 35, 36, 38, 41], surgery time (*n* = 15) [8, 18, 20, 21, 22, 24, 30, 32, 33, 34, 35, 36, 38, 39, 41], hospital days (*n* = 10) [8, 18, 21, 24, 30, 32, 34, 35, 36, 41], VAS score (*n* = 12) [8, 18, 20, 21, 22, 30, 32, 33, 34, 35, 38, 41], Cobb angle (*n* = 7) [21, 32, 33, 34, 35, 36, 38] as well as incidence of complications (*n* = 8) [21, 22, 24, 32, 34, 35, 36, 38]. Sixteen articles were cohort studies [18, 20, 21, 22, 24, 29, 30, 31, 32, 35, 36, 37, 38, 39, 40, 41], and three studies were RCTs [8, 21, 33].

**TABLE 1:** The main characteristics of included studies. The quality assessment refers to the NOS score for cohort studies. RCTs are presented in Section 3.3.2.

First author	Year	Nationality	Study design	Intervention	Sample size (male) (*n*)	Age (years)	Average follow‐up time (months)	Outcomes	Quality assessment
Yahui Gong [3]	2017	China	RCT	TFPSF	35 (24)	37.01 ± 6.83	12	^c^, ^d^, ^e^	Not applicable
				CPPSF	35 (23)	36.89 ± 7.21	12		
Irfanullah Shah [30]	2022	Saudi Arabia	Cohort study	TFPSF	22 (15)	43.95 ± 7.93	6	^b^, ^c^, ^d^, ^e^	6
				CPPSF	22 (16)	45.95 ± 8.87	6		
Marco G. A. Teli [31]	2021	UK	Cohort study	TFPSF	26 (23)	38	24	^b^	7
				CPPSF	31 (28)	41	24		
Pengfa Tu [21]	2022	China	Cohort study	TFPSF	25 (15)	42.60 ± 4.20	6	^b^, ^c^, ^d^, ^e^, ^f^, ^g^	6
				CPPSF	25 (13)	45.10 ± 6.40	6		
Yafei Xu [32]	2023	China	Cohort study	TFPSF	49 (23)	57.97 ± 12.82	14.23	^b^, ^c^, ^d^, ^e^, ^f^, ^g^	7
				CPPSF	49 (21)	57.34 ± 12.64	14.23		
Yang Liu [33]	2023	China	RCT	TFPSF	32	43.3 ± 3.2	NA	^b^, ^c^, ^e^, ^f^	Not applicable
				CPPSF	32	43.3 ± 3.2	NA		
Ming Yang [34]	2018	China	RCT	TFPSF	30 (17)	41.45 ± 10.01	15.4	^b^, ^c^, ^d^, ^e^, ^f^, ^g^	Not applicable
				CPPSF	30 (14)	39.90 ± 9.89	15.4		
JingYao Ye [35]	2022	China	Cohort study	TFPSF	21 (15)	55.43 ± 14.83	33.81	^b^, ^c^, ^d^, ^e^, ^f^, ^g^	7
				CPPSF	26 (21)	58.12 ± 15.72	33.81		
N. Bronsard [22]	2013	France	Cohort study	TFPSF	30 (21)	43.5 ± 12.8	25.86	^b^, ^c^, ^e^, ^g^	7
				CPPSF	30 (12)	40.4 ± 12.3	24.57		
Sheng‐yang Du [36]	2022	China	Cohort study	CPPSF	36 (26)	39.8 ± 10.7	12	^a^, ^b^, ^c^, ^d^, ^f^, ^g^	7
				RPPSF	34 (25)	42.8 ± 8.7	12		
Xu‐Qi Hu [20]	2023	China	Cohort study	TFPSF	24 (13)	53.92 ± 5.48	12	^a^, ^b^, ^c^, ^f^	7
				RPPSF	26 (14)	58.04 ± 9.15	12		
Gennadiy A. Katsevman [37]	2021	USA	Cohort study	CPPSF	20 (15)	57.3 ± 17.3	NA	^a^	7
				RPPSF	17 (9)	61.4 ± 13.8	NA		
Yongjun Li [38]	2023	China	Cohort study	CPPSF	40 (19)	43 ± 6.3	6	^a^, ^b^, ^c^, ^e^, ^f^, ^g^	6
				RPPSF	45 (22)	42 ± 7.9	6		
Shu Lin [24]	2022	China	Cohort study	CPPSF	61 (28)	51.49 ± 10.73	8	^a^, ^b^, ^c^, ^d^, ^g^	8
				RPPSF	65 (31)	50.21 ± 9.21	8		
Hao Liu [39]	2017	China	Cohort study	CPPSF	23 (15)	43.2 ± 14.2	6	^a^, ^c^	6
				OPPSF	30 (19)	41.6 ± 17.9	6		
Jianhua Lu [40]	2020	China	Cohort study	CPPSF	52 (29)	53.4 ± 15.1	NA	^a^	7
				OPPSF	45 (24)	50.2 ± 13.9	NA		
Peng Yang [18]	2020	China	Cohort study	TFPSF	36 (23)	49.3 ± 11.2	27.8	^a^, ^b^, ^c^, ^d^, ^e^	8
				OPPSF	36 (25)	48.7 ± 9.7	28.2		
Ren‐Jie Zhang [29]	2022	China	Cohort study	TFPSF	30 (22)	45.03 ± 11.80	NA	^a^	6
				CPPSF	14 (6)	41.64 ± 17.96	NA		
				RPPSF	30 (20)	49.13 ± 10.19	NA		
Xu Zhu [41]	2023	China	Cohort study	TFPSF	48 (30)	45.3 ± 9.0	15.8	^a^, ^b^, ^c^, ^d^, ^e^	8
				CPPSF	39 (25)	48.8 ± 12.8	15.9		
				OPPSF	43 (36)	45.8 ± 9.2	16.3		

Abbreviations: CPPSF, conventional fluoroscopy (C‐arm) percutaneous pedicle screw fixation; OPPSF, O‐arm assisted percutaneous pedicle screw fixation; RPPSF, robot‐assisted percutaneous pedicle screw fixation; TFPSF, traditional freehand pedicle screw fixation.

^a^ Accuracy rate of PSP.

^b^ IBL.

^c^ Surgery time.

^d^ Hospital days.

^e^ VAS score.

^f^ Cobb angle.

^g^ The incidence of complications.

### 3.3 Quality Assessment

#### 3.3.1 Cohort Studies

According to the NOS, among the 16 cohort studies, five were rated 6 points, eight were rated 7 points, and three were rated 8 points, indicating an overall moderate‐to‐high quality of the studies. Notably, these studies demonstrated a low risk of bias in terms of population selection and intergroup comparability. However, limitations were observed in the blinding of outcome assessment, control of confounding factors, follow‐up duration, and the completeness of outcome data, which were the main reasons for the deduction of points.

#### 3.3.2 RCTs

**FIGURE 2:**
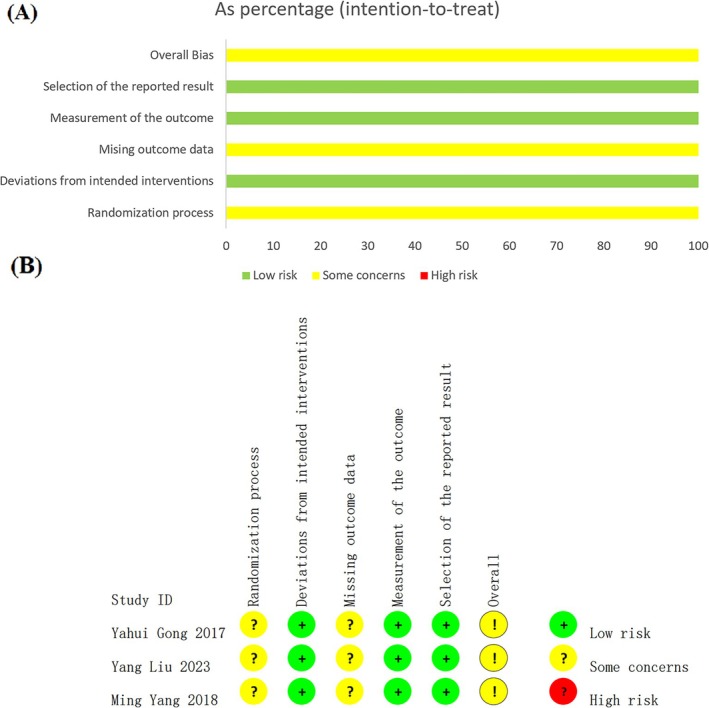
(A) Risk of bias summary, (B) risk of bias graph.

According to RoB 2.0, all three RCTs were rated as “some concerns” (Figure 2). Specifically, the studies had a low risk of bias in deviations from intended interventions, measurement of the outcome, and selection of the reported result. In the randomization process, all three studies only mentioned “randomization” without further describing the randomization methods, resulting in a rating of “some concerns.” Furthermore, in the domain of missing outcome data, none of the studies provided information regarding the extent of missing outcome data and thereby were rated as “some concerns.”

### 3.4 NMA

#### 3.4.1 Primary Outcomes

##### 3.4.1.1 Accuracy Rate of PSP

**FIGURE 3:**
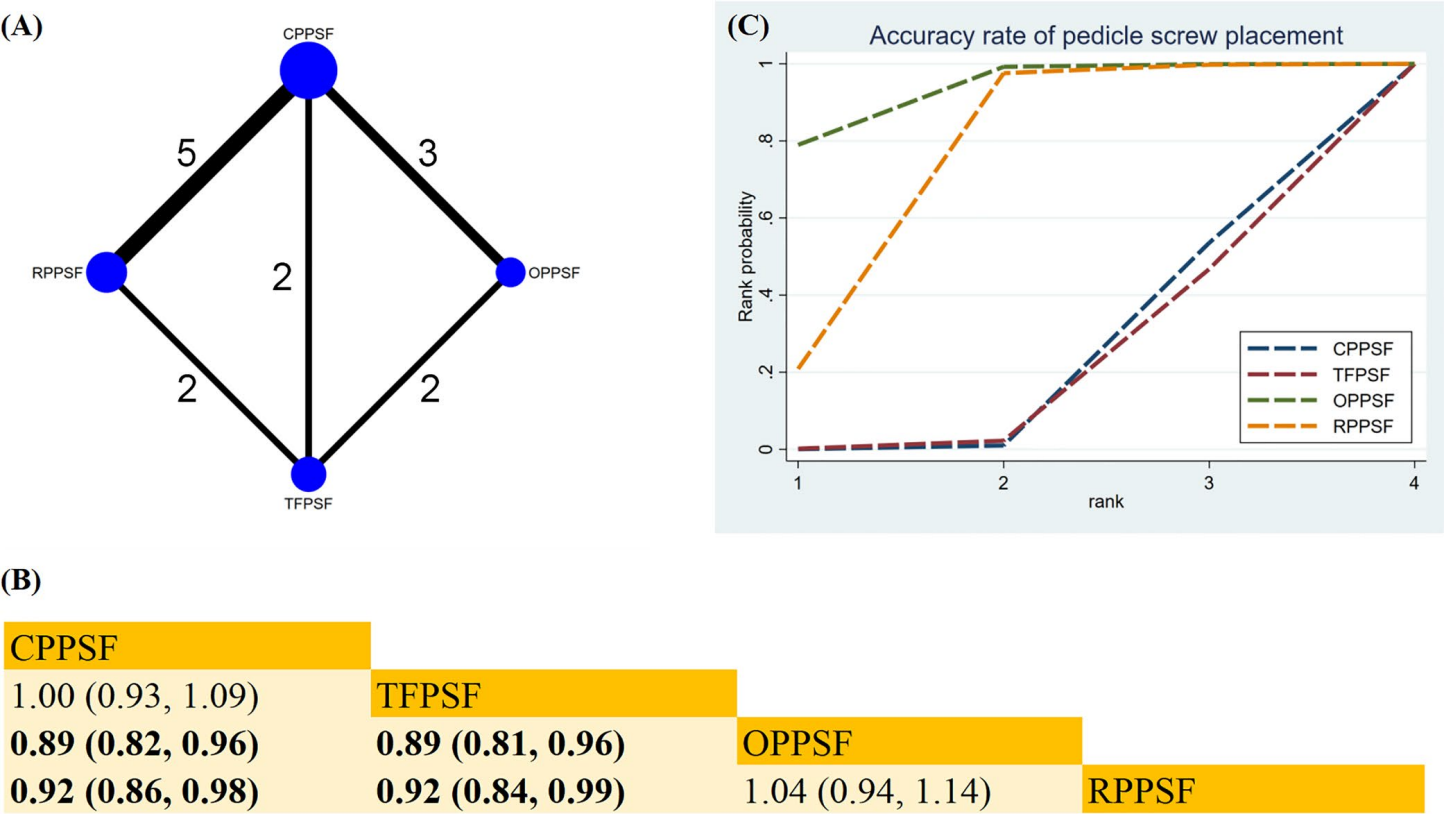
Network plot, SUCRA curve, and comparative outcomes of NMA. (A) Network plot for the accuracy rate of PSP. (B) Relative effects of different surgical approaches on the accuracy rate of PSP. (C) SUCRA graph of the accuracy rate of PSP. Estimates are expressed as RR with 95% CrI. The numbers adjacent to the connecting lines in the network plot indicate the number of studies that directly compared the two corresponding interventions. Comparisons between surgical approaches should be read from left to right. Statistically significant results are highlighted in bold.

Ten studies examined the accuracy rate of PSP (Figure 3A). The main findings of our NMA are presented in Figure 3B. In contrast to OPPSF, both CPPSF (RR 0.89; 95% CrI [0.82, 0.96]) and TFPSF (RR 0.89; 95% CrI [0.81, 0.96]) demonstrated lower accuracy. Similarly, when compared to RPPSF, both CPPSF (RR 0.92; 95% CrI [0.86, 0.98]) and TFPSF (RR 0.92; 95% CrI [0.84, 0.99]) exhibited lower accuracy rates. According to the SUCRA analysis, OPPSF emerged as the most effective surgical approach in improving accuracy (SUCRA = 92.7%) (Figure 3C) (Table S1).

##### 3.4.1.2 IBL

**FIGURE 4:**
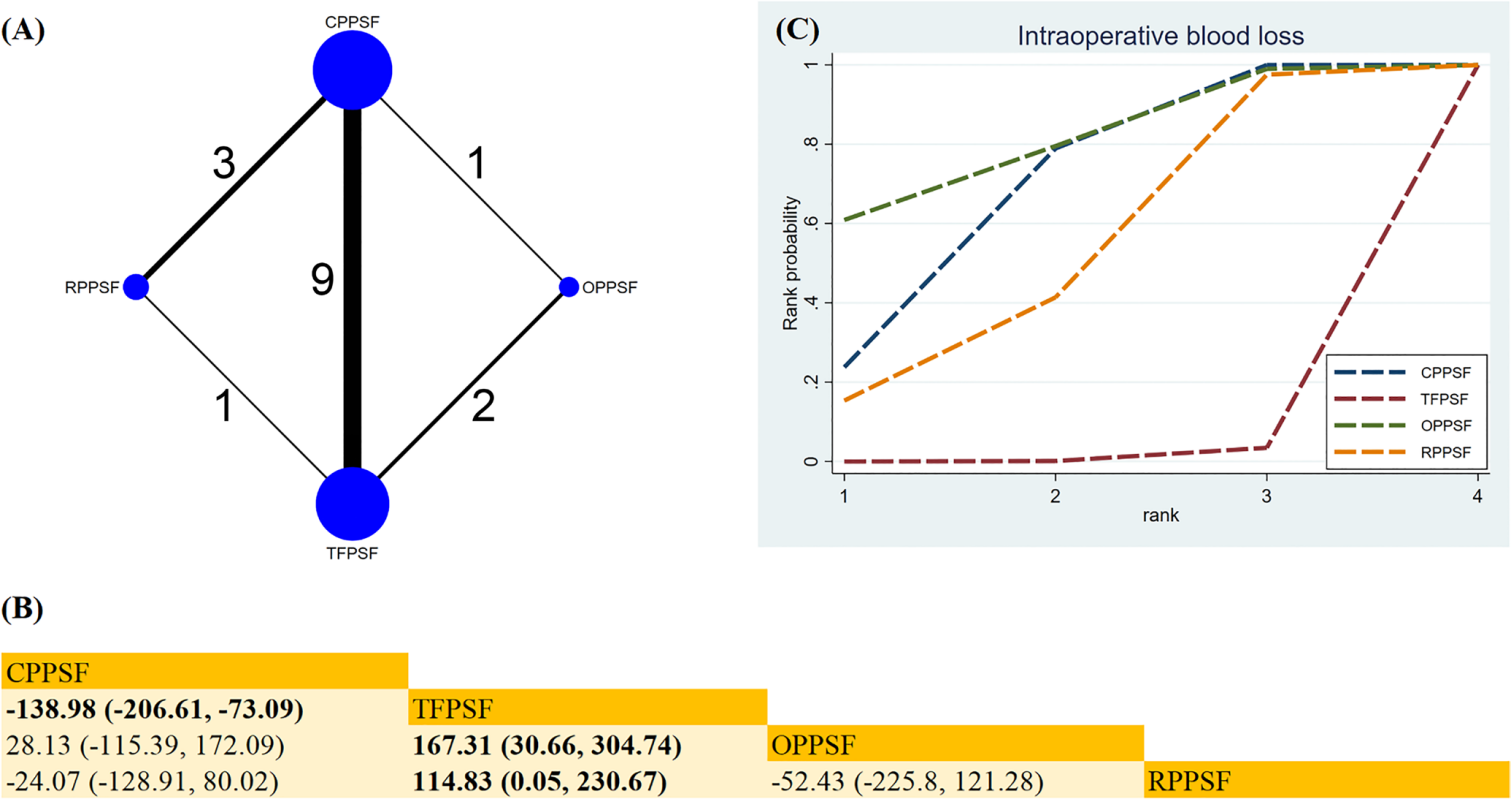
Network plot, SUCRA curve, and comparative outcomes of NMA. (A) Network plot for IBL. (B) Relative effects of different surgical approaches on IBL. (C) SUCRA graph of IBL. Estimates are expressed as MD with 95% CrI. The numbers adjacent to the connecting lines in the network plot indicate the number of studies that directly compared the two corresponding interventions. Comparisons between surgical approaches should be read from left to right. Statistically significant results are highlighted in bold.

Four methods across 14 studies were analyzed to assess intraoperative blood loss (IBL) (Figure 4A). CPPSF (MD −138.98; 95% CrI [−206.61, −73.09]), OPPSF (MD −167.31; 95% CrI [−304.74, −30.66]), and RPPSF (MD −114.83; 95% CrI [−230.67, −0.05]) significantly reduced IBL compared with TFPSF (Figure 4B). Regarding SUCRA, OPPSF emerged as the most effective surgical approach for minimizing blood loss (SUCRA = 79.8%) (Figure 4C) (Table S1).

#### 3.4.2 Secondary Outcomes

##### 3.4.2.1 Surgery Time

**FIGURE 5:**
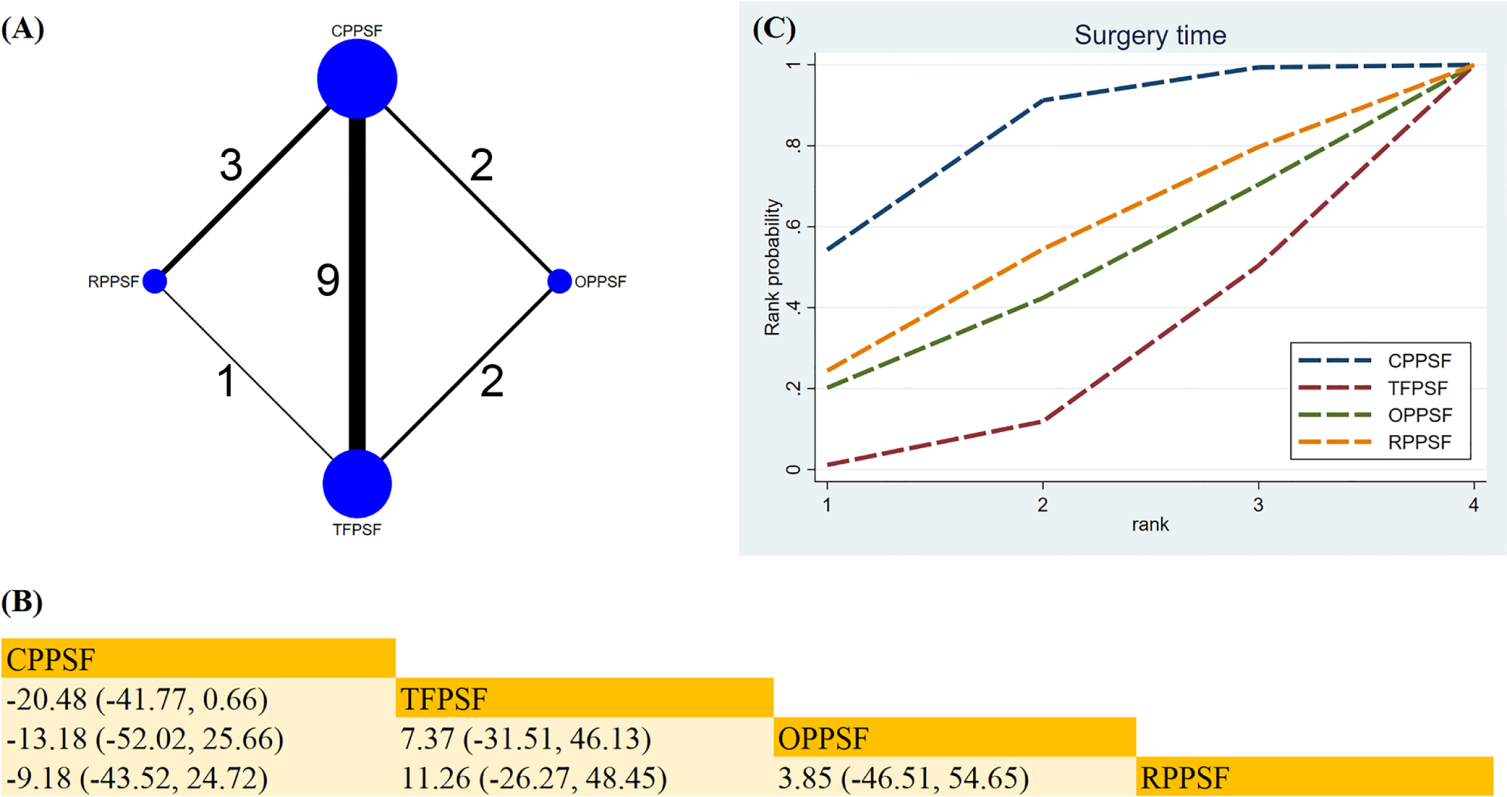
Network plot, SUCRA curve, and comparative outcomes of NMA. (A) Network plot for surgery time. (B) Relative effects of different surgical approaches on surgery time. (C) SUCRA graph of surgery time. Estimates are shown in MD with 95% CrI. The numbers adjacent to the connecting lines in the network plot indicate the number of studies that directly compared the two corresponding interventions. Comparisons between surgical approaches should be interpreted from left to right. Statistically significant results are highlighted in bold.

A total of 15 studies reported surgery time (Figure 5A). Pairwise comparisons across groups revealed no statistically significant differences in the data (Figure 5B). The ranking based on SUCRA indicated CPPSF as the most effective surgical approach for reducing surgery time (SUCRA = 81.6%) (Figure 5C) (Table S1).

##### 3.4.2.2 Hospital Days

**FIGURE 6:**
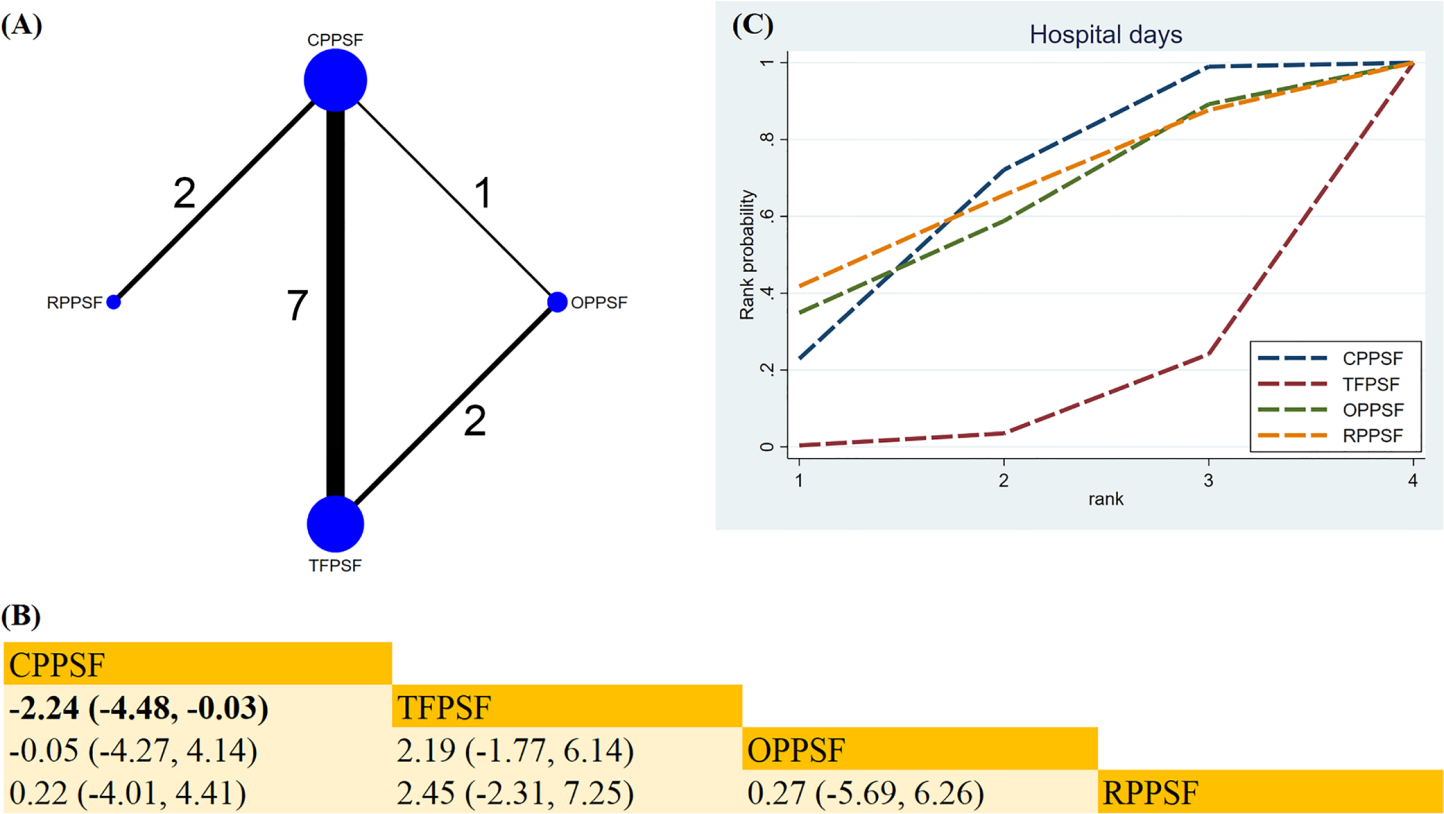
Network plot, SUCRA curve, and comparative outcomes of NMA. (A) Network plot for hospital days. (B) Relative effects of different surgical approaches on hospital days. (C) SUCRA graph of hospital days. Estimates are shown in MD with 95% CrI. The numbers adjacent to the connecting lines in the network plot indicate the number of studies that directly compared the two corresponding interventions. Comparisons between surgical approaches should be interpreted from left to right. Statistically significant results are highlighted in bold.

The data from 10 studies comparing four surgical methods provided information on the respective lengths of hospitalization (Figure 6A). When compared to TFPSF, CPPSF significantly reduced the number of hospital days in thoracolumbar fracture patients (MD −2.24; 95% CrI [−4.48, −0.03]) (Figure 6B). The SUCRA revealed that RPPSF was the most effective surgical approach for reducing the hospital days (SUCRA = 65.0%) (Figure 6C) (Table S1).

##### 3.4.2.3 VAS Score

**FIGURE 7:**
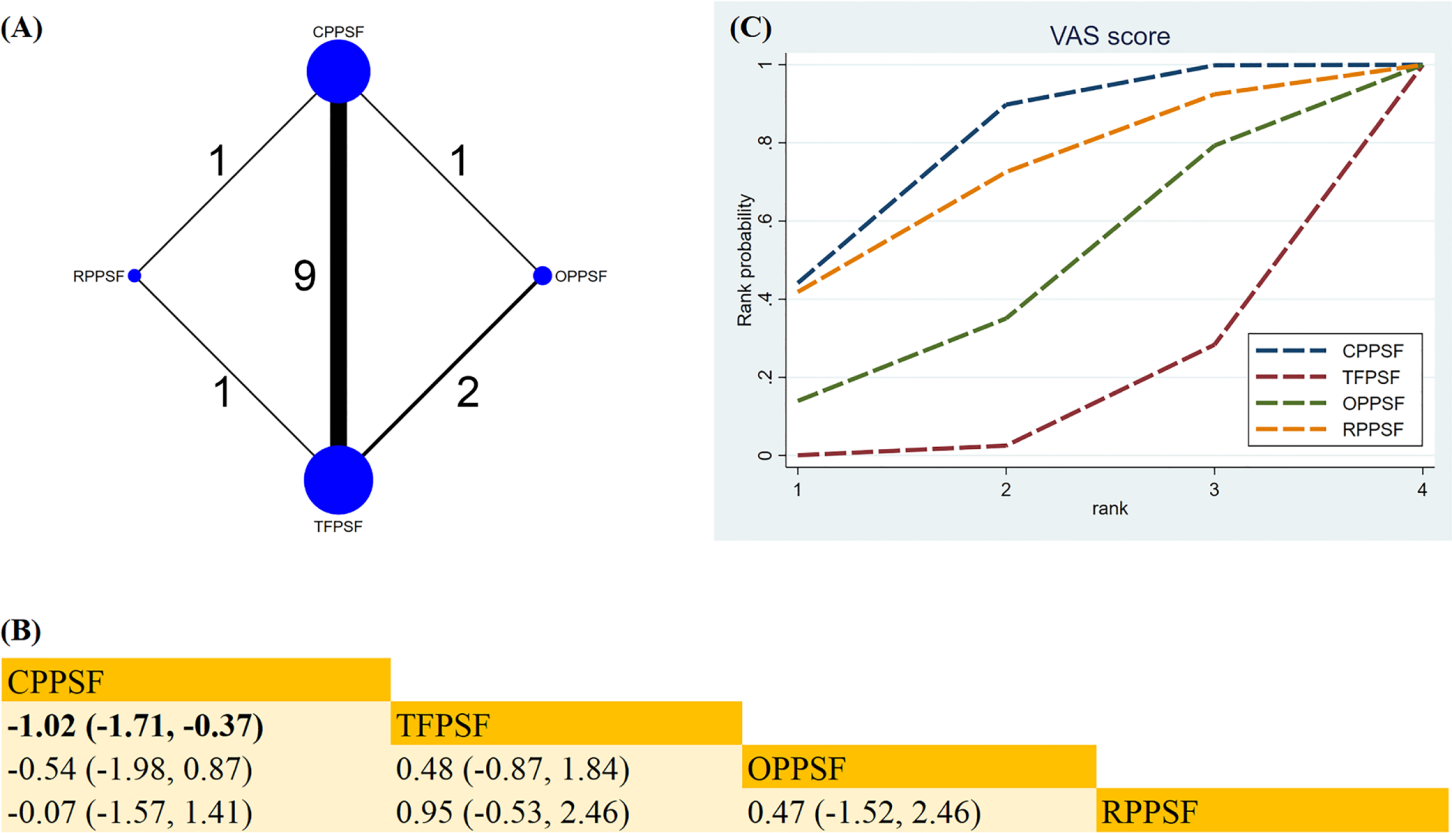
Network plot, SUCRA curve, and comparative outcomes of NMA. (A) Network plot for VAS score. (B) Relative effects of different surgical approaches on VAS score. (C) SUCRA graph of VAS score. Estimates are shown in MD with 95% CrI. The numbers adjacent to the connecting lines in the network plot indicate the number of studies that directly compared the two corresponding interventions. Comparisons between surgical approaches should be interpreted from left to right. Statistically significant results are highlighted in bold.

Twelve studies evaluated the four surgical approaches in terms of VAS scores (Figure 7A). Compared with TFPSF, CPPSF significantly reduced the VAS score (MD −1.02; 95% CrI [−1.71, −0.37]) (Figure 7B). Based on the SUCRA, CPPSF was likely the best surgical approach to reduce the VAS score (SUCRA = 77.9%) (Figure 7C) (Table S1).

##### 3.4.2.4 Cobb Angle

**FIGURE 8:**
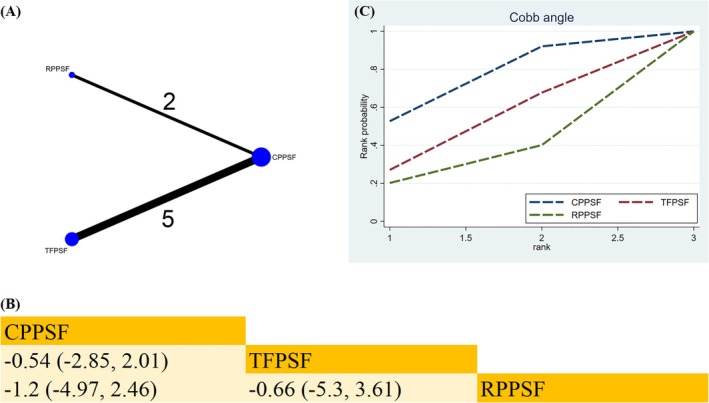
Network plot, SUCRA curve, and comparative outcomes of NMA. (A) Network plot for Cobb angle. (B) Relative effects of various surgical approaches on the Cobb angle. (C) SUCRA graph of Cobb angle. Estimates are shown in MD with 95% CrI. The numbers adjacent to the connecting lines in the network plot indicate the number of studies that directly compared the two corresponding interventions. Comparisons between surgical approaches should be interpreted from left to right. Statistically significant results are highlighted in bold.

Three methods (TFPSF, CPPSF, RPPSF) from seven studies were selected for Cobb angle analysis (Figure 8A). No statistically significant differences existed between the methods when compared pairwise (Figure 8B). Based on SUCRA, CPPSF was the best surgical option for reducing the Cobb angle (SUCRA = 72.4%) (Figure 8C) (Table S1).

##### 3.4.2.5 Incidence of Complications

**FIGURE 9:**
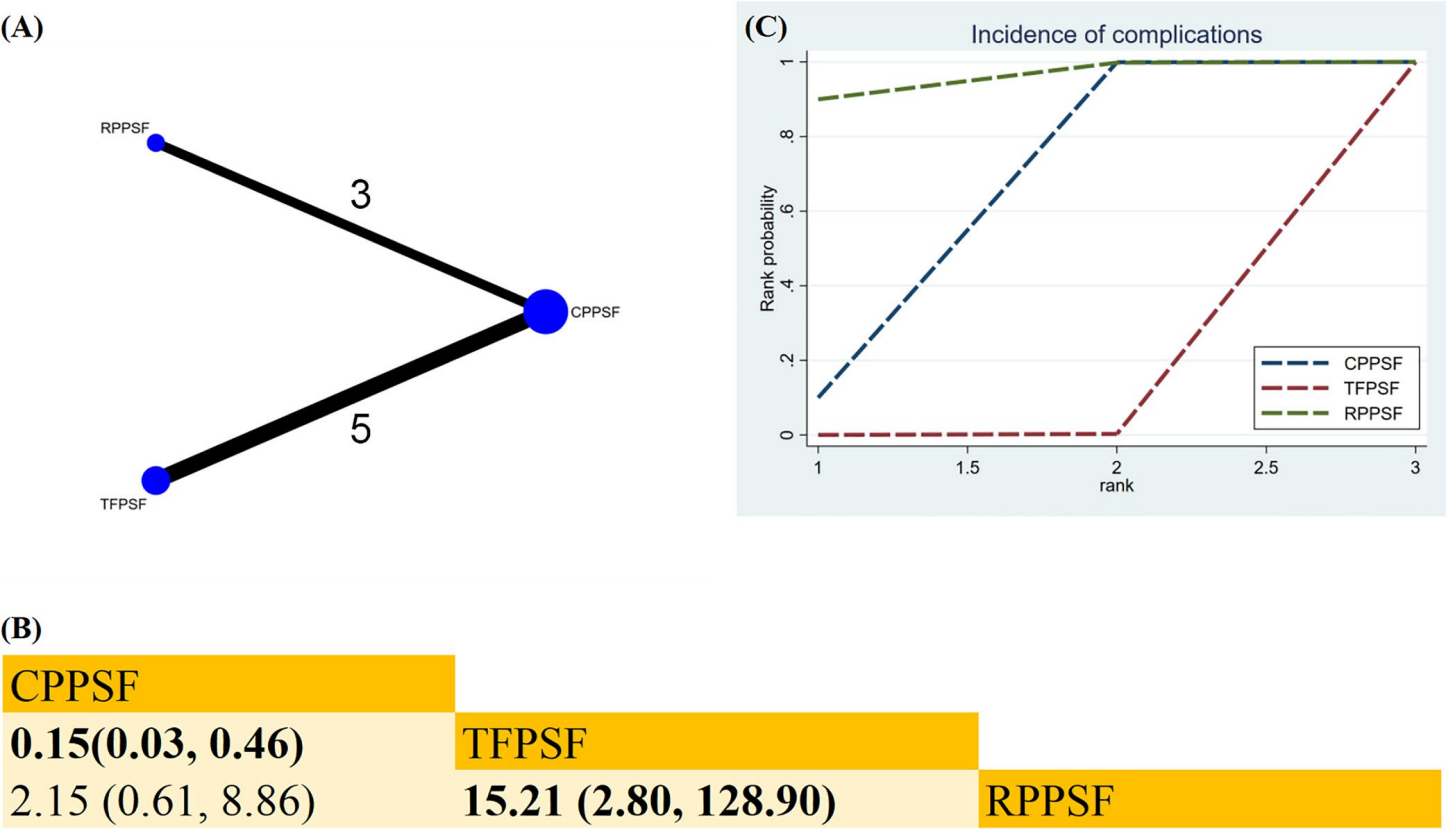
Network plot, SUCRA curve, and comparative outcomes of NMA. (A) Network plot for incidence of complications. (B) Relative effects of various surgical approaches on the incidence of complications. (C) SUCRA graph of the incidence of complications. Estimates are expressed as RR with 95% CrI. The numbers adjacent to the connecting lines in the network plot indicate the number of studies that directly compared the two corresponding interventions. Comparisons between surgical approaches should be read from left to right. Statistically significant results are highlighted in bold.

Eight studies provided data comparing the incidence of complications across three methods (TFPSF, CPPSF, RPPSF) (Figure 9A). Compared with TFPSF, both CPPSF (RR 0.15; 95% CrI [0.03, 0.46]) and RPPSF (RR 0.07; 95% CrI [0.01, 0.36]) significantly lowered the incidence of complications (Figure 9B). According to the SUCRA, RPPSF was the most effective approach in reducing the incidence of complications (SUCRA = 94.9%) (Figure 9C) (Table S1).

### 3.5 Consistency and Publication Bias Assessment

Good consistency was found when the consistency and inconsistency models were compared through DIC. All closed‐loop models had DIC changes of less than five. An analysis of local consistency was conducted for five outcome measures: screw placement accuracy, IBL, surgical duration, length of hospitalization, and VAS score. At a 95% CI, all *p* values were higher than 0.05, indicating that local consistency was achieved (Table S2).

Based on the comparison‐adjusted funnel plots, there was no publication bias (Figures [Link], [Link]).

### 3.6 Sensitivity Analyses

#### 3.6.1 Dominant Country Analysis

In the dominant country analysis, 15 of 19 included studies (78.9%) originated from China [8, 18, 20, 21, 24, 29, 32, 33, 34, 35, 36, 38, 39, 40, 41]. The China‐specific sensitivity analysis revealed notable deviations from the primary findings. For IBL, the significant difference between TFPSF and RPPSF identified in the primary analysis (MD 114.83, 95% CrI [0.05, 230.67]) was no longer statistically significant (MD 85.66, 95% CrI [−23.9, 194.92]). Regarding hospitalization duration, a previously nonsignificant difference between RPPSF and TFPSF emerged as statistically significant in favor of RPPSF (primary analysis MD −2.45, 95% CrI [−7.25, 2.31]; sensitivity analysis MD −3.29, 95% CrI [−6.43, −0.28]). All other statistically significant pairwise comparisons and SUCRA‐based top‐ranked interventions remained unchanged. Notably, the Cobb angle was not reanalyzed, as all included studies for this outcome were conducted in China; thus, the results remained identical to those of the primary analysis. Full data are provided in Figures [Link], [Link] and Table S3.

#### 3.6.2 High‐Quality Study Analysis

Eight studies met the predefined high‐quality criteria, with only IBL, VAS scores, and operative duration meeting the minimum sample size requirements for sensitivity analyses [18, 20, 22, 31, 32, 35, 36, 41]. In comparison to the primary analysis, previously significant differences in IBL lost statistical significance for both TFPSF versus OPPSF (primary analysis MD 167.31, 95% CrI [30.66, 304.74]; sensitivity analysis MD 162.32, 95% CrI [−6.94, 333.68]) and TFPSF versus RPPSF (primary analysis MD 114.83, 95% CrI [0.05, 230.67]; sensitivity analysis MD 78.38, 95% CrI [−109.52, 268.98]). For VAS scores, the evidence network remained incomplete due to the absence of a direct comparison between OPPSF and RPPSF. Additionally, the TFPSF–CPPSF difference became nonsignificant (primary analysis MD 1.02, 95% CrI [0.37, 1.17]; sensitivity analysis MD 0.48, 95% CrI [−0.07, 1.07]). The SUCRA hierarchy for VAS scores shifted, with RPPSF (87.8%) surpassing CPPSF (77.9%) as the top‐ranked intervention. Results of operative duration were consistent with those of the primary analysis, with no statistically significant pairwise differences, and CPPSF maintaining its position as the top‐ranked technique by SUCRA. Complete results are presented in Figures [Link], [Link] and Table S4.

## 4 Discussion

### 4.1 Main Findings

This NMA systematically compared the efficacy of four pedicle screw fixation techniques for thoracolumbar fractures. The comprehensive analysis of 19 studies involving 1344 patients demonstrated that minimally invasive techniques collectively outperformed conventional freehand methods, while exhibiting distinct outcome profiles across various efficacy measures. OPPSF was proven to be the best intervention for increasing PSP accuracy and reducing IBL. CPPSF appeared to exhibit better performance in reducing postoperative VAS scores and shortening hospital stays. RPPSF demonstrated potential advantages in minimizing surgical complications. For operative time and Cobb angle, the absence of statistical significance necessitates further investigation. These differential advantages of techniques provide evidence‐based guidance for individualized clinical decision‐making.

### 4.2 Primary Outcomes

The accuracy of PSP critically influences long‐term prognosis, while intraoperative hemorrhage represents a common surgical complication. Our findings, which rank OPPSF highest for both accuracy and blood loss control, are consistent with previous meta‐analyses [18, 40, 41, 42, 43, 44]. This superiority is primarily attributable to the O‐arm real‐time intraoperative 3D imaging capability. Unlike conventional C‐arm fluoroscopy, which provides 2D images, the O‐arm system generates high‐resolution 3D reconstructions that precisely delineate complex spinal anatomy, including pedicle morphology and spatial relationships with neurovascular structures [45]. This enhanced visualization enables surgical precision approaching direct visual guidance.

The system facilitates preoperative planning through simulated screw trajectory using multiplanar reconstructed images, allowing precise assessment of insertion depth and angulation, which are the key determinants of placement accuracy. Intraoperatively, real‐time visualization of screw position relative to critical structures significantly reduces malposition risks and associated complications [46]. The combination of improved visual field clarity and minimized tissue dissection through smaller incisions collectively contributes to reduced blood loss. Furthermore, the O‐arm's imaging capability permits immediate identification and management of hemorrhagic events.

In contrast, C‐arm provides limited 2D guidance that proves particularly inadequate in complex cases involving spinal deformity or anomalous anatomy, where image misinterpretation may lead to surgical errors and increased bleeding risk. Consequently, OPPSF emerges as the preferred approach for cases demanding exceptional precision and minimal blood loss, including complex fracture patterns (AO type B/C) [47], fractures with vascular compromise [48], and revision surgeries with distorted anatomy.

### 4.3 Secondary Outcomes

Secondary outcome analyses revealed more nuanced technical characteristics. Regarding postoperative VAS scores, CPPSF attained the highest ranking with a SUCRA value of 77.9%, exhibiting a significant reduction in comparison to TFPSF. Previous meta‐analyses have shown that, compared to TFPSF, CPPSF can improve patients' VAS scores, with this difference being statistically significant, which aligns with our findings [35]. As CPPSF is a relatively mature technique with widespread application and performed by many highly skilled surgeons, its associated pain levels may be slightly lower than those of the newer technique group. Additionally, due to the minimally invasive nature and shorter operative duration of CPPSF, its pain levels are also expected to be lower than those of TFPSF. However, sensitivity analyses excluding lower‐quality studies indicated that RPPSF emerged as the top‐ranked intervention (SUCRA = 87.8%), while CPPSF declined to second (SUCRA = 60.9%), although these differences did not reach statistical significance in pairwise comparisons. This shift may reflect more rigorous outcome measurements (e.g., standardized timing for VAS assessment and blinding), increased surgical experience with RPPSF, or the conservative statistical approaches applied in higher‐quality studies. Overall, based on current evidence, CPPSF appears to be a reliable choice for pain management in standard clinical scenarios, while the potential superiority of RPPSF in pain control necessitates additional rigorous research.

In terms of hospitalization duration, CPPSF demonstrated statistically significant reductions compared to TFPSF, whereas RPPSF achieved the highest SUCRA ranking (65.0%). Prior meta‐analyses have also demonstrated that CPPSF significantly reduces hospital stays relative to TFPSF [32, 34], which aligns with the results of our analysis. Notably, sensitivity analyses excluding non‐Chinese studies revealed that the advantage of RPPSF over TFPSF became statistically significant, further supporting its high SUCRA ranking. Meanwhile, CPPSF maintained its superiority over TFPSF across all analyses. These results suggest that RPPSF protocols employed in China may emphasize earlier mobilization, and the earlier adoption of robotic surgery in China has likely contributed to the greater accumulated surgical expertise with this technique [49]. In light of these results, CPPSF seems to be universally effective for reducing hospitalization across practice settings, while the potential of RPPSF to achieve statistically significant reductions in hospital days outside China remains to be substantiated through additional researches.

Concerning the incidence of complications, the analysis included a comprehensive set of adverse events: wound infections, persistent pain, nerve injury, postoperative anemia, postoperative bronchitis, intraoperative fracture displacement, substantial IBL, lower limb deep vein thrombosis, and other related events. RPPSF achieved the highest SUCRA ranking (94.9%) for complication rates, with both CPPSF and RPPSF demonstrating statistically significant reductions in complication incidence compared to TFPSF. Previous work by Chen et al. similarly confirmed a statistically significant reduction in postoperative complications with RPPSF compared to TFPSF [50]. These findings imply that, when cost considerations are disregarded, opting for RPPSF may reduce complication rates, especially in high‐risk patient populations where safety is prioritized.

### 4.4 Inconclusive Outcomes

Regarding surgical duration and Cobb angle, the SUCRA results suggest that CPPSF is the optimal intervention (respectively 81.6% and 72.4%). However, the results for both did not reach statistical significance. A systematic review concerning surgical duration by Feng et al. also revealed statistically insignificant variance between CPPSF and other groups [42], which corroborates the viewpoint of our study. The lack of significant differences may reflect variations in surgical expertise, fracture characteristics, and instrumentation protocols. Notably, our results contrast with Zhang et al.'s meta‐analysis, which reported statistically significant superiority of CPPSF for Cobb angle correction [9]. This discrepancy may be explained by the limited number of RCTs included in the present analysis, compared to the 18 RCTs incorporated in Zhang et al., as well as inconsistencies in follow‐up duration across studies. While SUCRA provides probabilistic rankings and may reflect trends even in the absence of statistical significance, reliance on SUCRA rankings alone in the absence of confirmatory statistical evidence may be misleading. Therefore, these findings should be interpreted with caution, and further validation through high‐quality studies is recommended.

### 4.5 Strengths and Limitations

The strength of our study lies in that it is the first NMA comparing four pivotal surgical techniques for thoracolumbar fractures, encompassing both traditional and novel interventions. The inclusion of these technically representative approaches provides a comprehensive evaluation across the evolutionary spectrum of surgical management. Furthermore, this study includes both RCTs and cohort studies, allowing for direct and indirect comparisons to assess the differences among the four interventions across seven outcome measures. Rigorous study selection criteria and comprehensive literature search strategies ensured methodological robustness, further strengthened by performing two sensitivity analyses to verify result stability.

However, several limitations of this study should be acknowledged. First, geographical bias must be considered, as 15 out of the 19 included studies were conducted in China. While sensitivity analyses showed consistent results for most outcomes within this context, and standardized surgical principles suggest potential generalizability, the limited representation from other healthcare systems restricts definitive conclusions regarding global applicability, particularly for OPPSF and RPPSF.

Second, residual clinical heterogeneity persists despite random‐effects modeling and sensitivity analyses. Variables including surgeon experience, operative protocols, fracture severity classifications, and perioperative care pathways could not be fully adjusted for due to inconsistent reporting across primary studies. This limitation affects the external validity of our findings.

Third, imbalanced sample sizes between conventional (CPPSF) and novel (OPPSF/RPPSF) techniques may increase Type II error risk. Although Bayesian methods partially accommodate data sparsity through prior distributions, evidenced by wider CrIs, interpretations regarding newer techniques require caution due to limited statistical power.

Finally, the predominance of observational studies over RCTs introduces potential residual confounding and reduces evidence certainty compared to exclusively randomized evidence. Additionally, language restriction to English publications may introduce selection bias.

### 4.6 Future Directions

To advance the field, future investigations should prioritize multinational, prospective designs with multicenter participation to standardize long‐term follow‐up and increase sample sizes for newer techniques such as OPPSF and RPPSF. These efforts will facilitate robust comparative analyses and promote technological advancement. In addition, the development of standardized global reporting guidelines and core outcome sets for thoracolumbar fracture surgeries, along with unified documentation of surgical procedures, would improve study comparability and enhance the external validity of future research.

## 5 Conclusion

The present NMA indicates the superior overall performance of PPSF compared to TFPSF in most assessed outcomes. Specifically, OPPSF may be the preferred technique in cases necessitating high screw placement accuracy and minimal blood loss, especially in anatomically complex or high‐risk patients. CPPSF, as a well‐established and widely available technique, appears to be the most suitable choice for routine clinical cases, with advantages in reducing hospitalization duration and postoperative pain. RPPSF exhibits potential in minimizing complication rates; however, its adoption requires advanced surgical expertise and incurs higher costs, warranting individualized cost–benefit assessments. Future multicenter RCTs are essential to further elucidate the specific indications and long‐term outcomes of these surgical techniques.

## Author Contributions

**Yankun Zhu:** conceptualization, methodology, investigation, formal analysis, data curation, writing – original draft, writing – review and editing, visualization. **Shuaiqi Zhu:** investigation, writing – original draft, writing – review and editing, visualization. **Yanan Li:** methodology, investigation, formal analysis, data curation, writing – original draft, writing – review and editing, visualization. **Kun Wang:** resources, writing – review and editing, supervision, project administration. All authors had full access to the data in the study and take responsibility for the integrity of the data and the accuracy of the data analysis.

## Ethics Statement

The authors have nothing to report.

## Consent

The authors have nothing to report.

## Conflicts of Interest

The authors declare no conflicts of interest.

## Supporting information


**Figure S1:** Funnel plot of the accuracy rate of pedicle screw placement.

**Figure S2:** Funnel plot of intraoperative blood loss.

**Figure S3:** Funnel plot of surgery time.

**Figure S4:** Funnel plot of hospital days.

**Figure S5:** Funnel plot of VAS score.

**Figure S6:** Funnel plot of Cobb angle.

**Figure S7:** Funnel plot of incidence of complications.

**Figure S8:** Network plot, SUCRA curve, and comparative outcomes of the dominant country sensitivity analysis. (A) Network plot for the accuracy rate of PSP. (B) Relative effects of different surgical approaches on the accuracy rate of PSP. (C) SUCRA graph of the accuracy rate of PSP. Estimates are expressed as RR with 95% CrI. The numbers adjacent to the connecting lines in the network plot indicate the number of studies that directly compared the two corresponding interventions. Comparisons between surgical approaches should be read from left to right. Statistically significant results are highlighted in bold.

**Figure S9:** Network plot, SUCRA curve, and comparative outcomes of the dominant country sensitivity analysis. (A) Network plot for IBL. (B) Relative effects of different surgical approaches on IBL. (C) SUCRA graph of IBL. Estimates are expressed as MD with 95% CrI. The numbers adjacent to the connecting lines in the network plot indicate the number of studies that directly compared the two corresponding interventions. Comparisons between surgical approaches should be read from left to right. Statistically significant results are highlighted in bold.

**Figure S10:** Network plot, SUCRA curve, and comparative outcomes of the dominant country sensitivity analysis. (A) Network plot for surgery time. (B) Relative effects of different surgical approaches on surgery time. (C) SUCRA graph of surgery time. Estimates are shown in MD with 95% CrI. The numbers adjacent to the connecting lines in the network plot indicate the number of studies that directly compared the two corresponding interventions. Comparisons between surgical approaches should be interpreted from left to right. Statistically significant results are highlighted in bold.

**Figure S11:** Network plot, SUCRA curve, and comparative outcomes of the dominant country sensitivity analysis. (A) Network plot for hospital days. (B) Relative effects of different surgical approaches on hospital days. (C) SUCRA graph of hospital days. Estimates are shown in MD with 95% CrI. The numbers adjacent to the connecting lines in the network plot indicate the number of studies that directly compared the two corresponding interventions. Comparisons between surgical approaches should be interpreted from left to right. Statistically significant results are highlighted in bold.

**Figure S12:** Network plot, SUCRA curve, and comparative outcomes of the dominant country sensitivity analysis. (A) Network plot for VAS score. (B) Relative effects of different surgical approaches on VAS score. (C) SUCRA graph of VAS score. Estimates are shown in MD with 95% CrI. The numbers adjacent to the connecting lines in the network plot indicate the number of studies that directly compared the two corresponding interventions. Comparisons between surgical approaches should be interpreted from left to right. Statistically significant results are highlighted in bold.

**Figure S13:** Network plot, SUCRA curve, and comparative outcomes of the dominant country sensitivity analysis. (A) Network plot for incidence of complications. (B) Relative effects of various surgical approaches on the incidence of complications. (C) SUCRA graph of the incidence of complications. Estimates are expressed as RR with 95% CrI. The numbers adjacent to the connecting lines in the network plot indicate the number of studies that directly compared the two corresponding interventions. Comparisons between surgical approaches should be read from left to right. Statistically significant results are highlighted in bold.

**Figure S14:** Network plot, SUCRA curve, and comparative outcomes of the high‐quality study sensitivity analysis. (A) Network plot for IBL. (B) Relative effects of different surgical approaches on IBL. (C) SUCRA graph of IBL. Estimates are expressed as MD with 95% CrI. The numbers adjacent to the connecting lines in the network plot indicate the number of studies that directly compared the two corresponding interventions. Comparisons between surgical approaches should be read from left to right. Statistically significant results are highlighted in bold.

**Figure S15:** Network plot, SUCRA curve, and comparative outcomes of the high‐quality study sensitivity analysis. (A) Network plot for surgery time. (B) Relative effects of different surgical approaches on surgery time. (C) SUCRA graph of surgery time. Estimates are shown in MD with 95% CrI. The numbers adjacent to the connecting lines in the network plot indicate the number of studies that directly compared the two corresponding interventions. Comparisons between surgical approaches should be interpreted from left to right. Statistically significant results are highlighted in bold.

**Figure S16:** Network plot, SUCRA curve, and comparative outcomes of the high‐quality study sensitivity analysis. (A) Network plot for VAS score. (B) Relative effects of different surgical approaches on VAS score. (C) SUCRA graph of VAS score. Estimates are shown in MD with 95% CrI. The numbers adjacent to the connecting lines in the network plot indicate the number of studies that directly compared the two corresponding interventions. Comparisons between surgical approaches should be interpreted from left to right. Statistically significant results are highlighted in bold.

**Data S1:** Retrieval strategy.

**Data S2:** PRISMA checklist.

**Table S1:** SUCRA values of four surgical techniques according to outcomes.

**Table S2:** Results of local inconsistency analysis.

**Table S3:** SUCRA values of four surgical techniques according to outcomes in the dominant country sensitivity analysis.

**Table S4:** SUCRA values of four surgical techniques according to outcomes in the high‐quality study sensitivity analysis.

## Data Availability

The data that supports the findings of this study are available in the supplementary material of this article.
