# Supplementary figures and images for: Robot‐Assisted, Conventional Fluoroscopy (C‐Arm), O‐Arm Navigation, and Freehand Pedicle Screw Fixation in Thoracolumbar Spine Fracture Surgery: A Network Meta‐Analysis
Source: Orthop Surg. 2025 Oct 11;17(12):3302–17. doi: 10.1111/os.70189 (PMC12685484; doi:10.1111/os.70189)

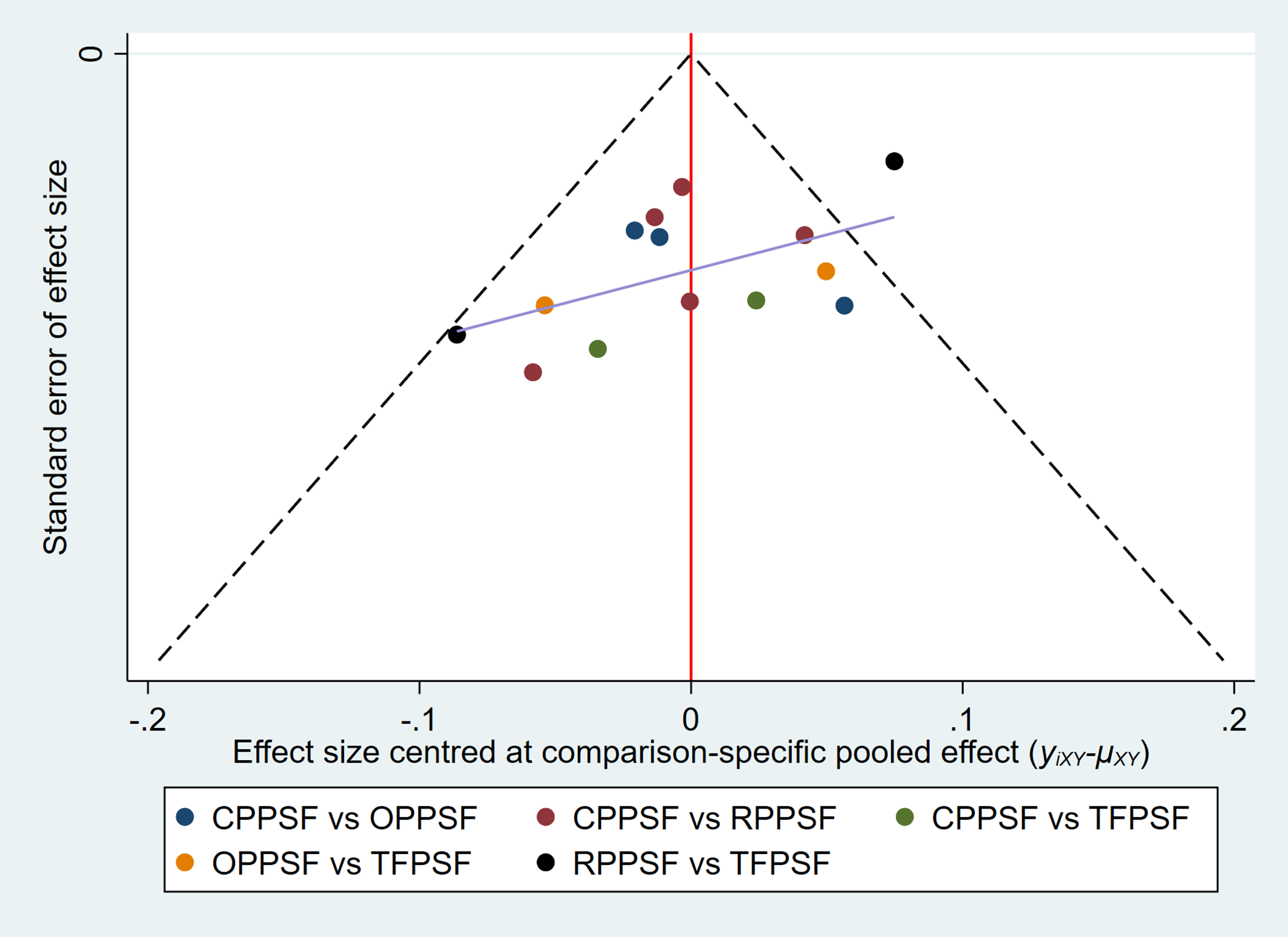

Supplement: Supplementary file 1 — Figure S1: Funnel plot of the accuracy rate of pedicle screw placement. [file OS-17-3302-s006.tif]

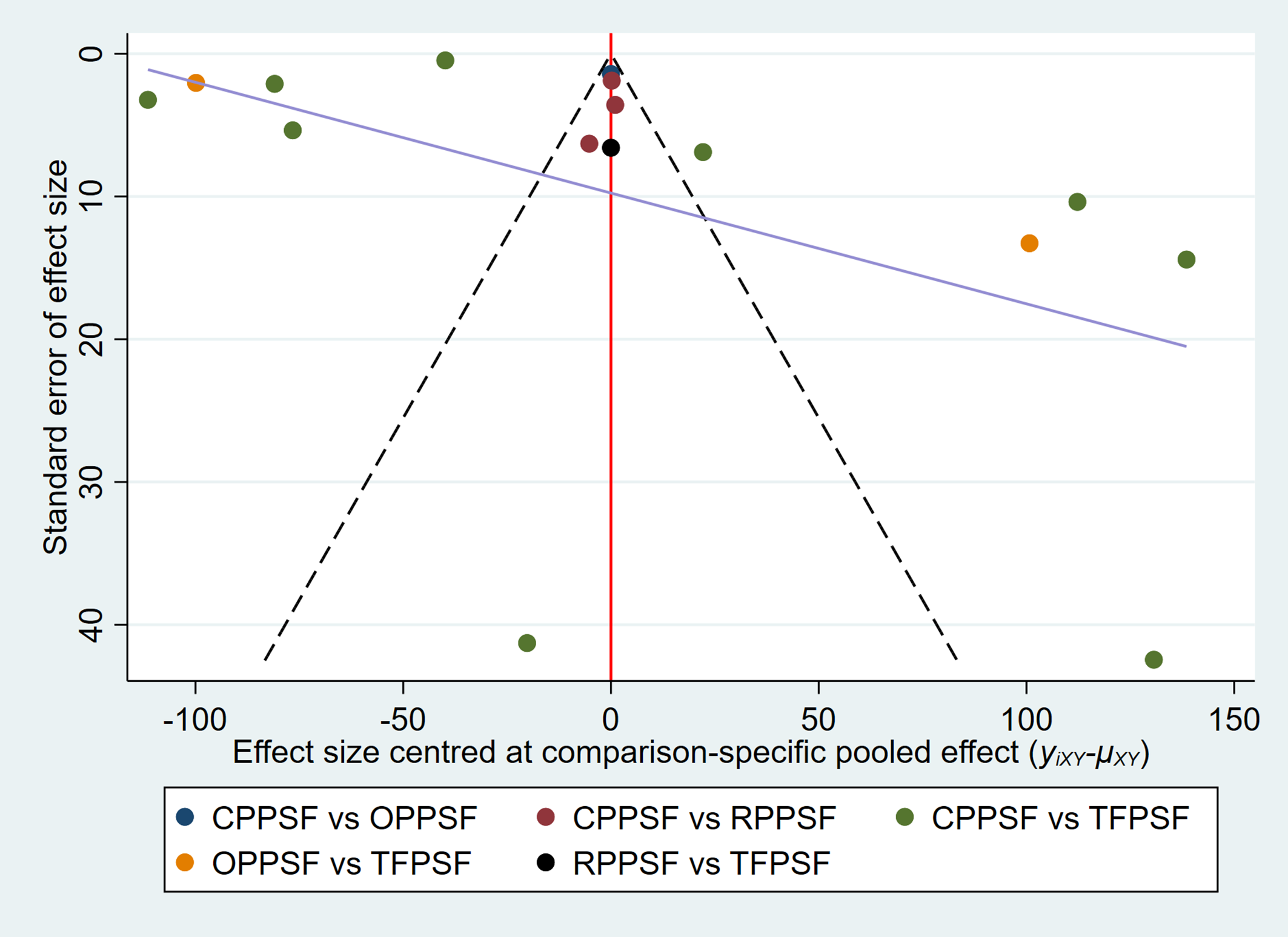

Supplement: Supplementary file 2 — Figure S2: Funnel plot of intraoperative blood loss. [file OS-17-3302-s011.tif]

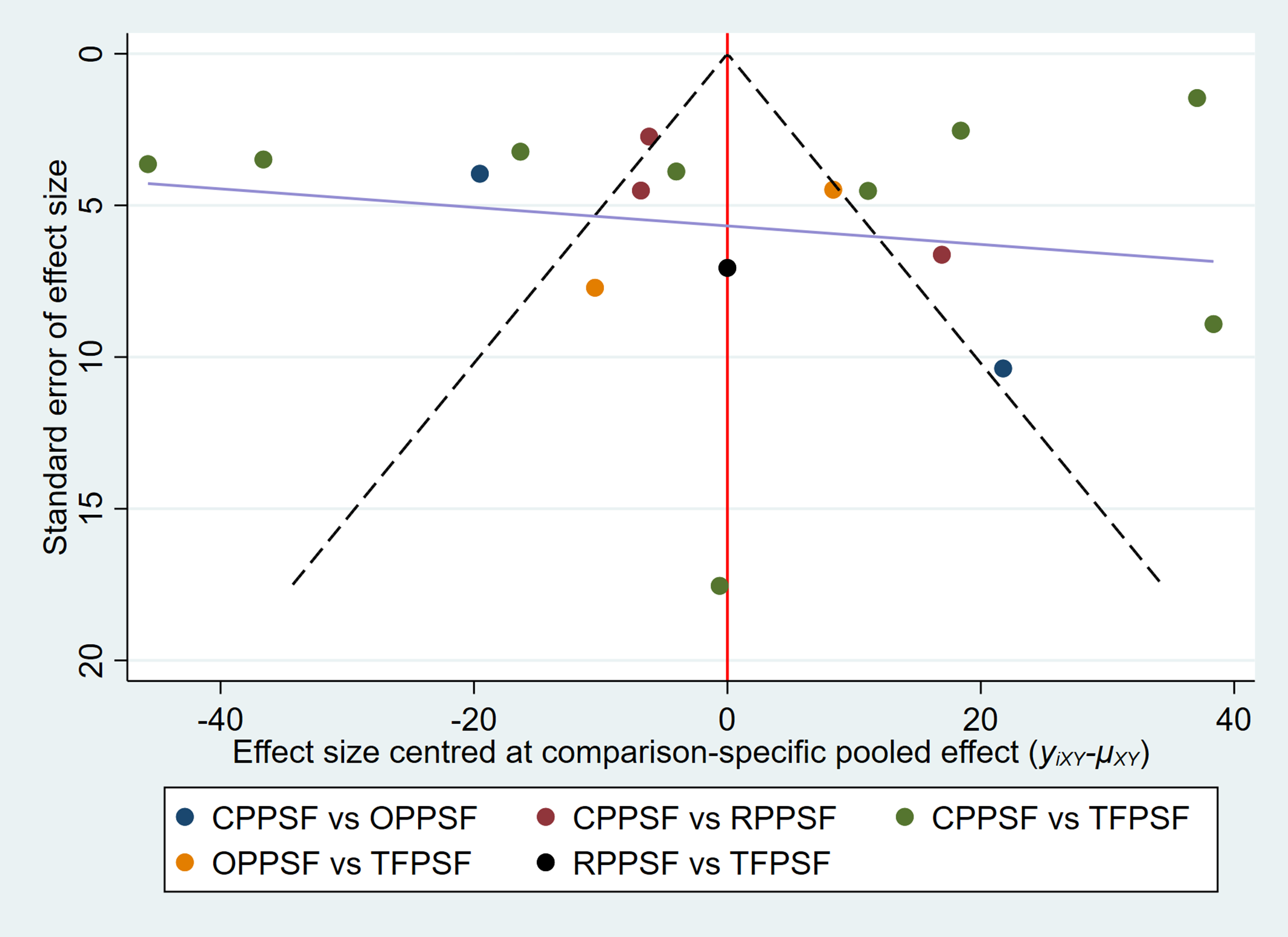

Supplement: Supplementary file 3 — Figure S3: Funnel plot of surgery time. [file OS-17-3302-s010.tif]

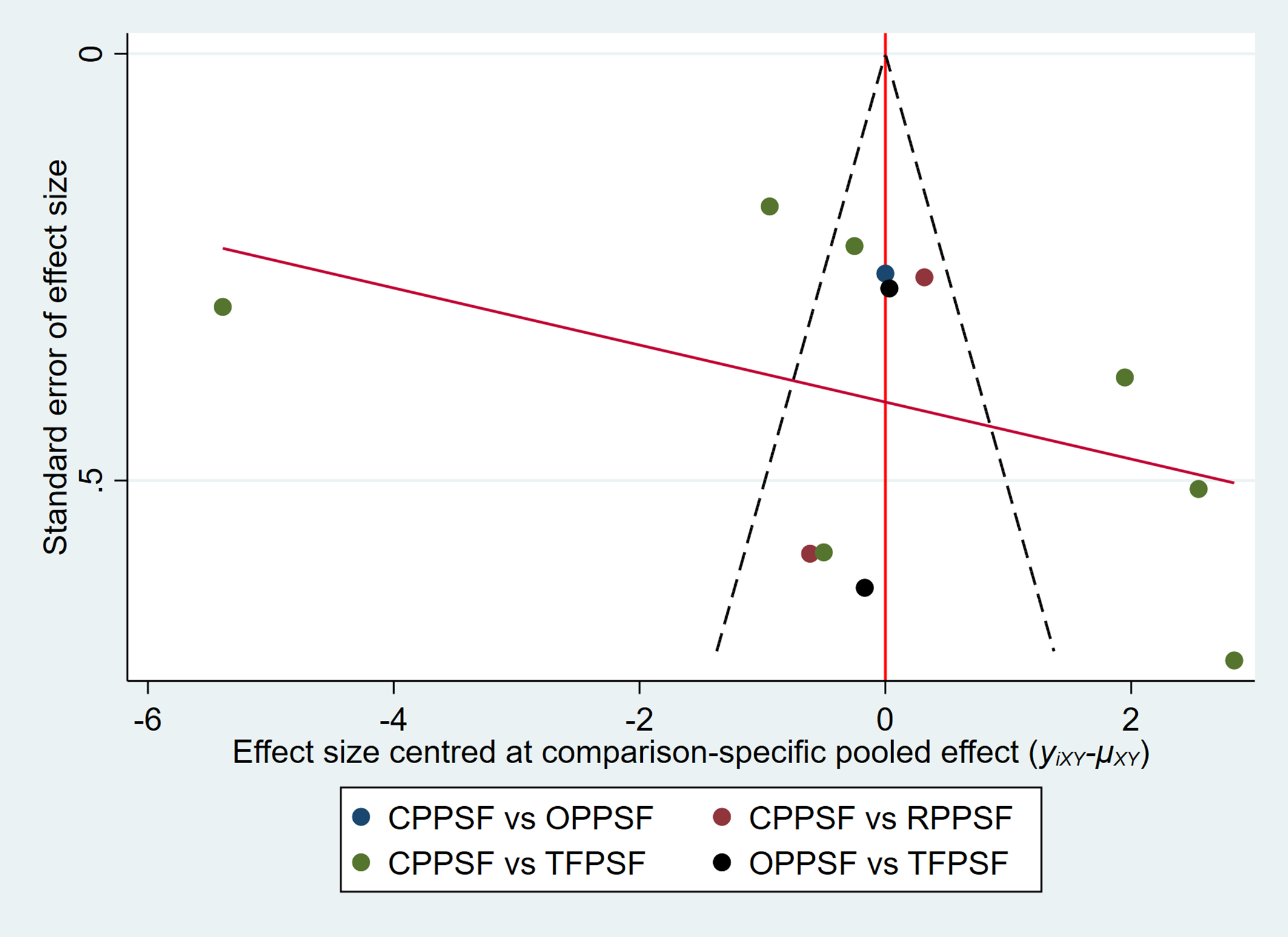

Supplement: Supplementary file 4 — Figure S4: Funnel plot of hospital days. [file OS-17-3302-s022.tif]

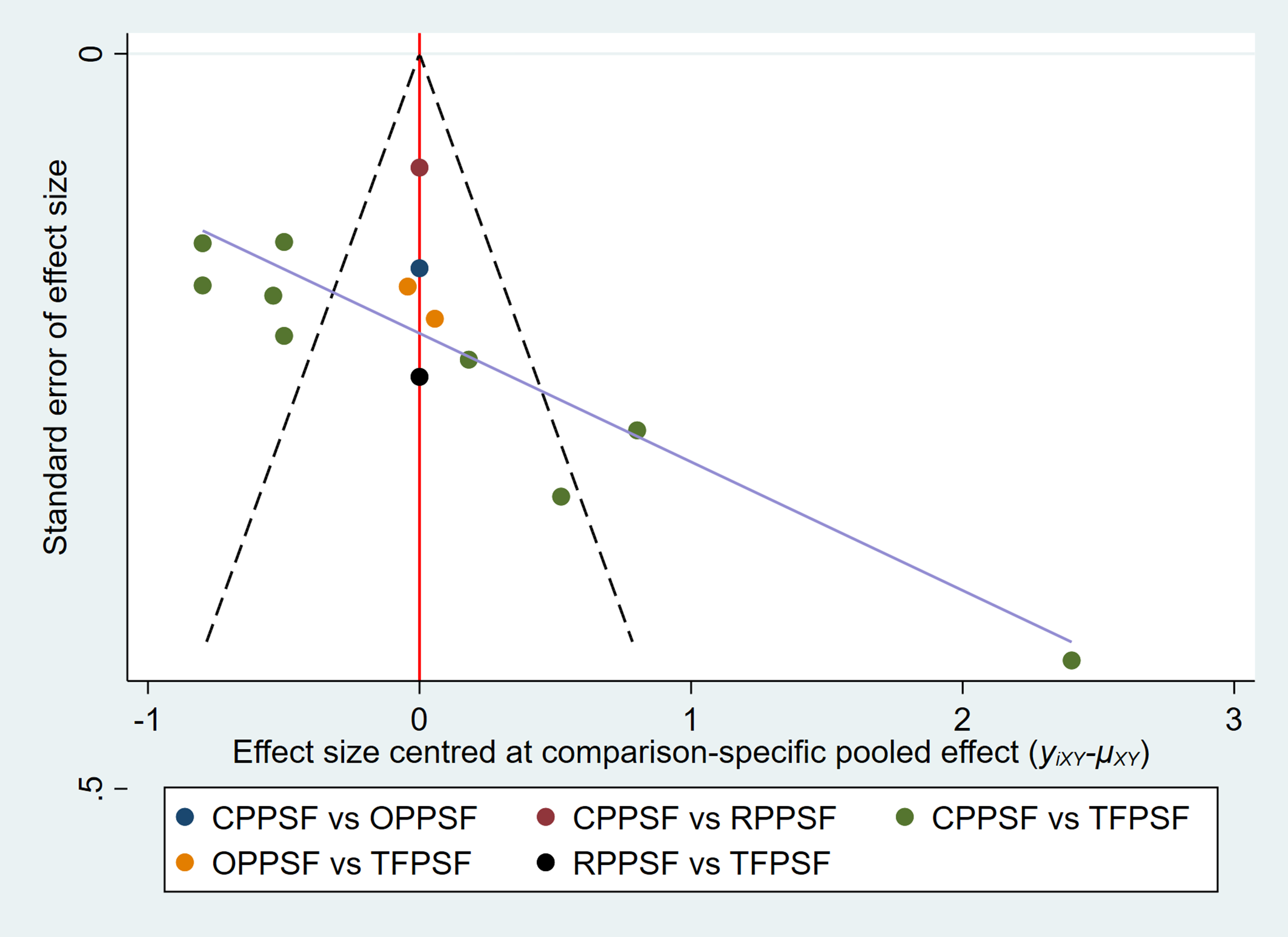

Supplement: Supplementary file 5 — Figure S5: Funnel plot of VAS score. [file OS-17-3302-s002.tif]

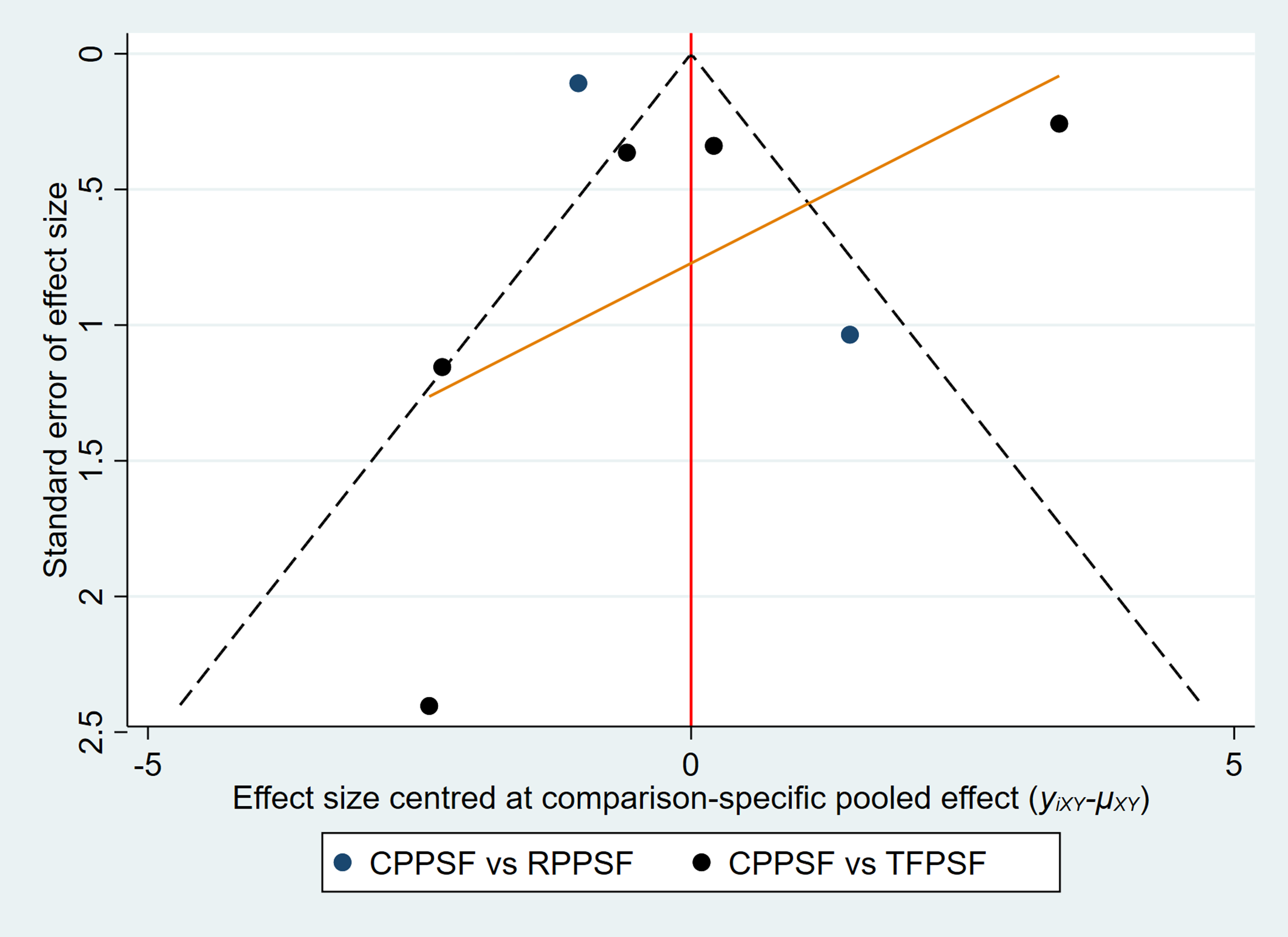

Supplement: Supplementary file 6 — Figure S6: Funnel plot of Cobb angle. [file OS-17-3302-s019.tif]

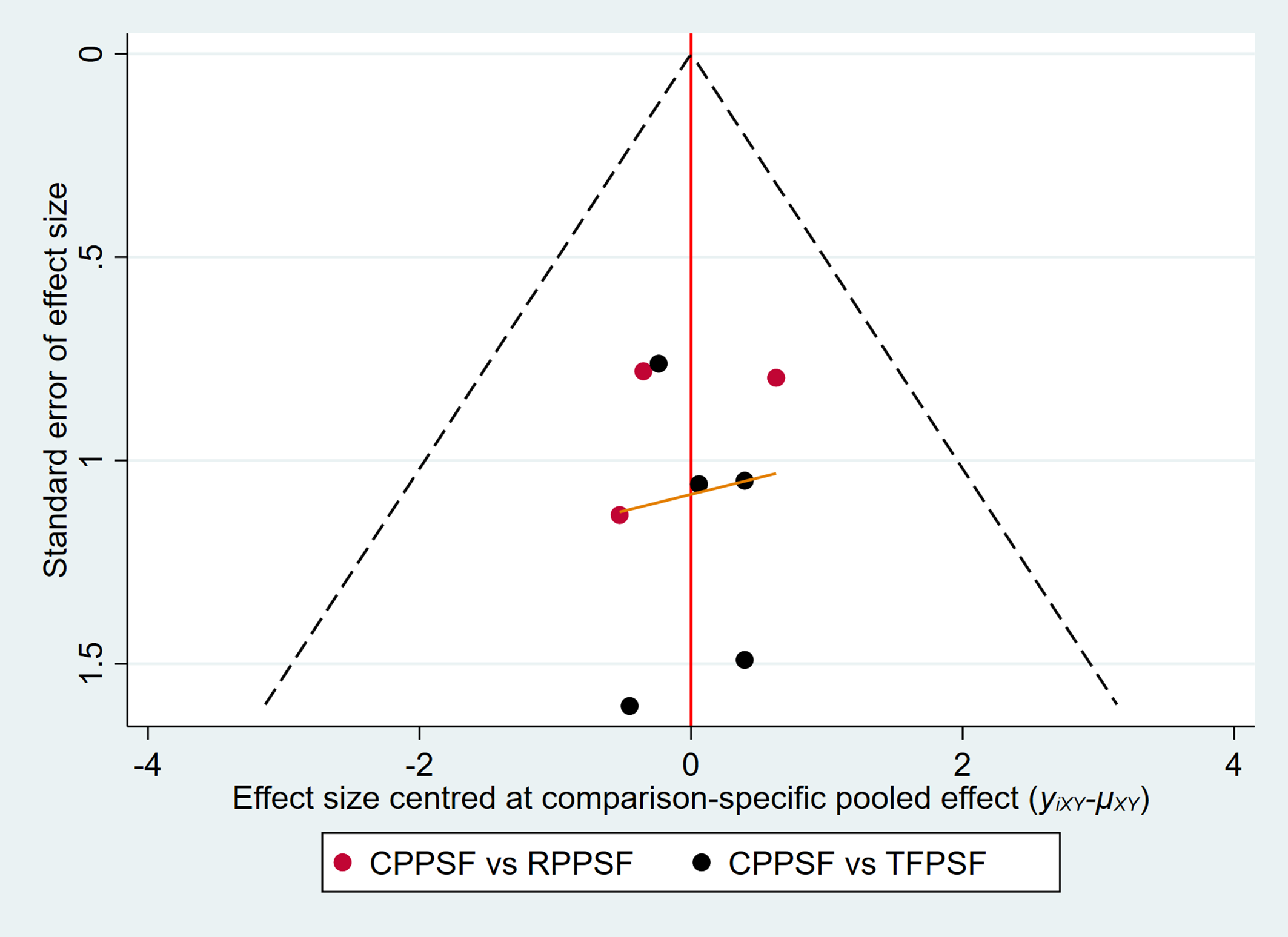

Supplement: Supplementary file 7 — Figure S7: Funnel plot of incidence of complications. [file OS-17-3302-s004.tif]

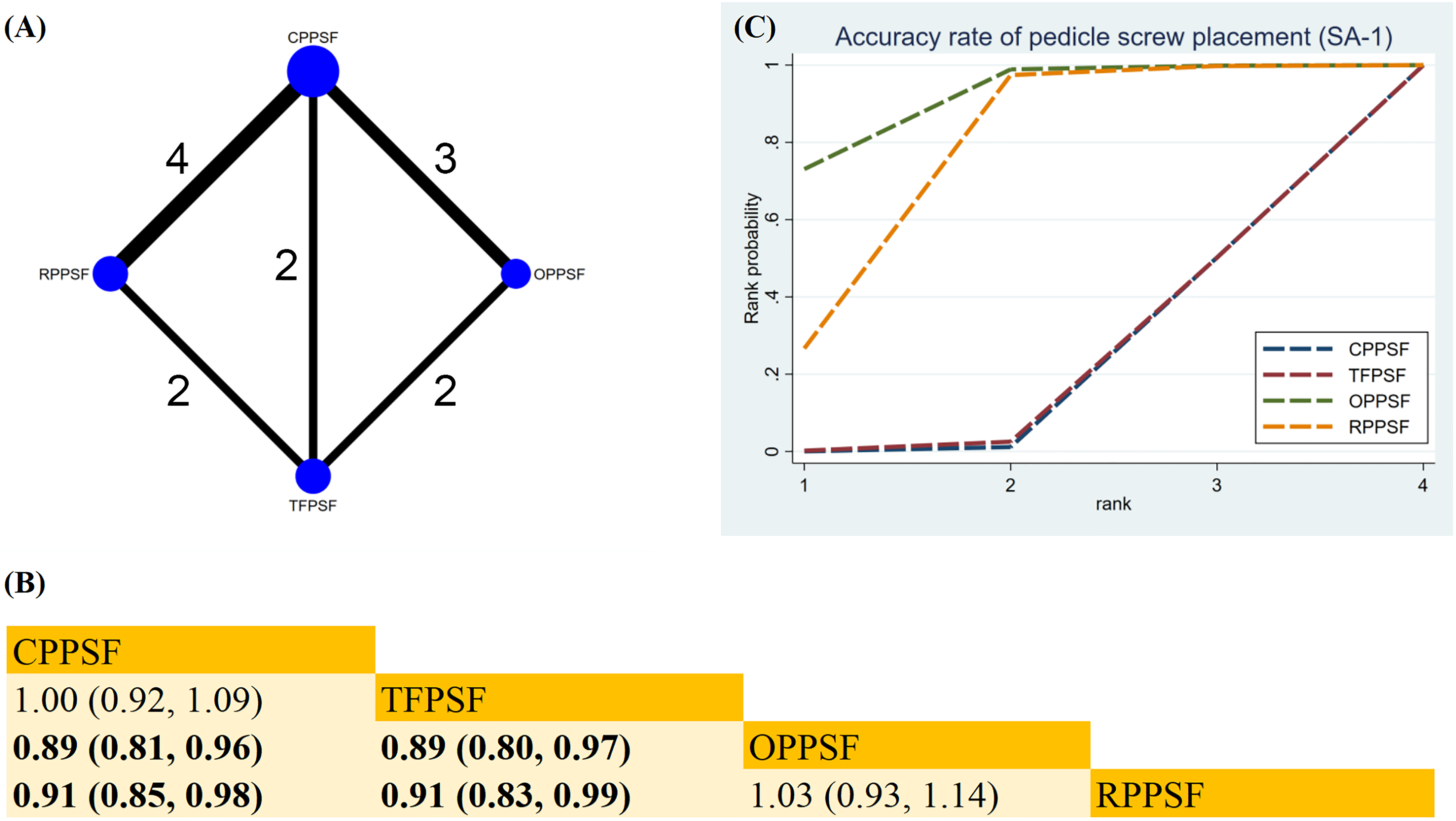

Supplement: Supplementary file 8 — Figure S8: Network plot, SUCRA curve, and comparative outcomes of the dominant country sensitivity analysis. (A) Network plot for the accuracy rate of PSP. (B) Relative effects of different surgical approaches on the accuracy rate of PSP. (C) SUCRA graph of the accuracy rate of PSP. Estimates are expressed as RR with 95% CrI. The numbers adjacent to the connecting lines in the network plot indicate the number of studies that directly compared the two corresponding interventions. Comparisons between surgical approaches should be read from left to right. Statistically significant results are highlighted in bold. [file OS-17-3302-s003.tif]

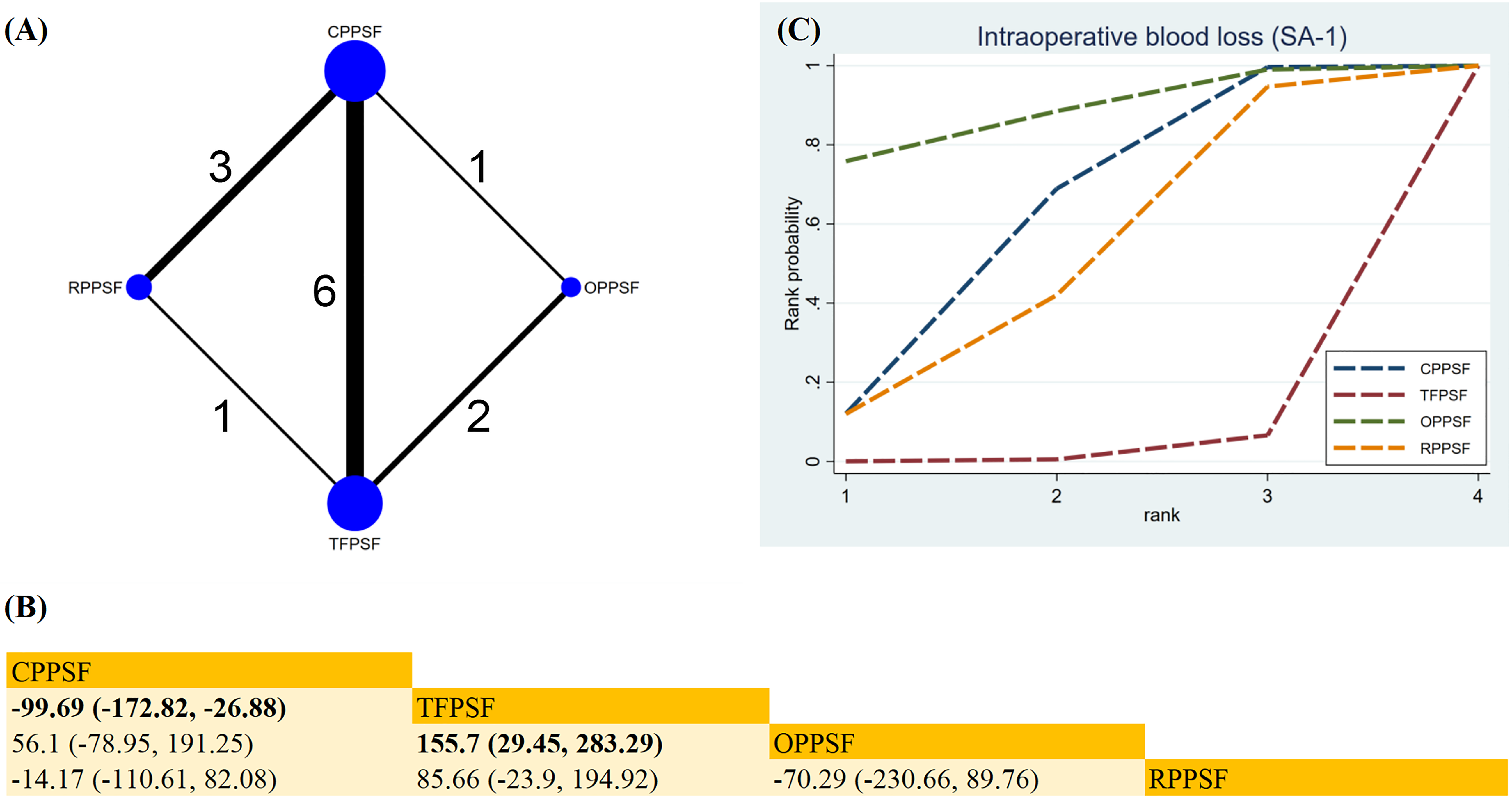

Supplement: Supplementary file 9 — Figure S9: Network plot, SUCRA curve, and comparative outcomes of the dominant country sensitivity analysis. (A) Network plot for IBL. (B) Relative effects of different surgical approaches on IBL. (C) SUCRA graph of IBL. Estimates are expressed as MD with 95% CrI. The numbers adjacent to the connecting lines in the network plot indicate the number of studies that directly compared the two corresponding interventions. Comparisons between surgical approaches should be read from left to right. Statistically significant results are highlighted in bold. [file OS-17-3302-s020.tif]

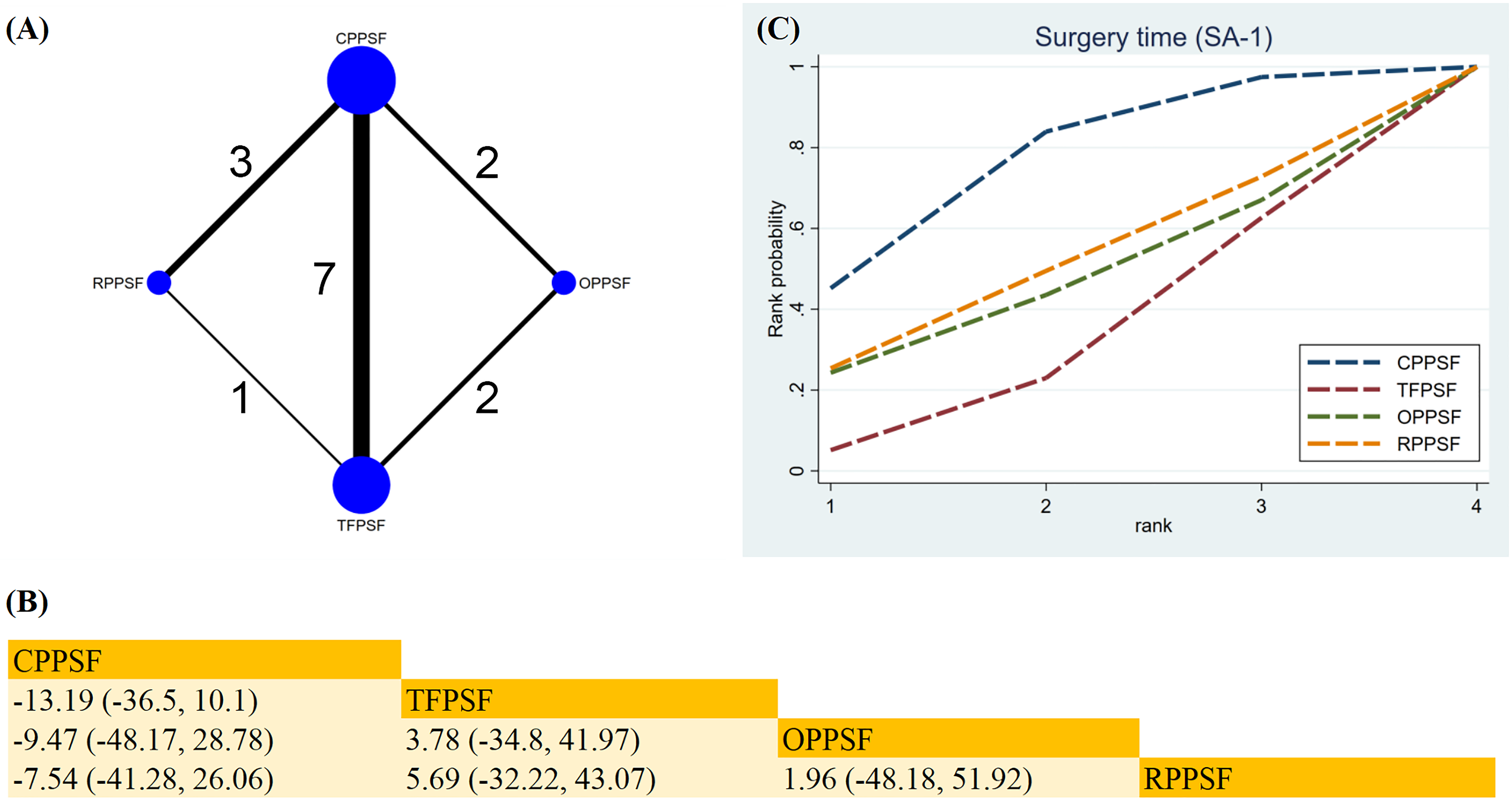

Supplement: Supplementary file 10 — Figure S10: Network plot, SUCRA curve, and comparative outcomes of the dominant country sensitivity analysis. (A) Network plot for surgery time. (B) Relative effects of different surgical approaches on surgery time. (C) SUCRA graph of surgery time. Estimates are shown in MD with 95% CrI. The numbers adjacent to the connecting lines in the network plot indicate the number of studies that directly compared the two corresponding interventions. Comparisons between surgical approaches should be interpreted from left to right. Statistically significant results are highlighted in bold. [file OS-17-3302-s005.tif]

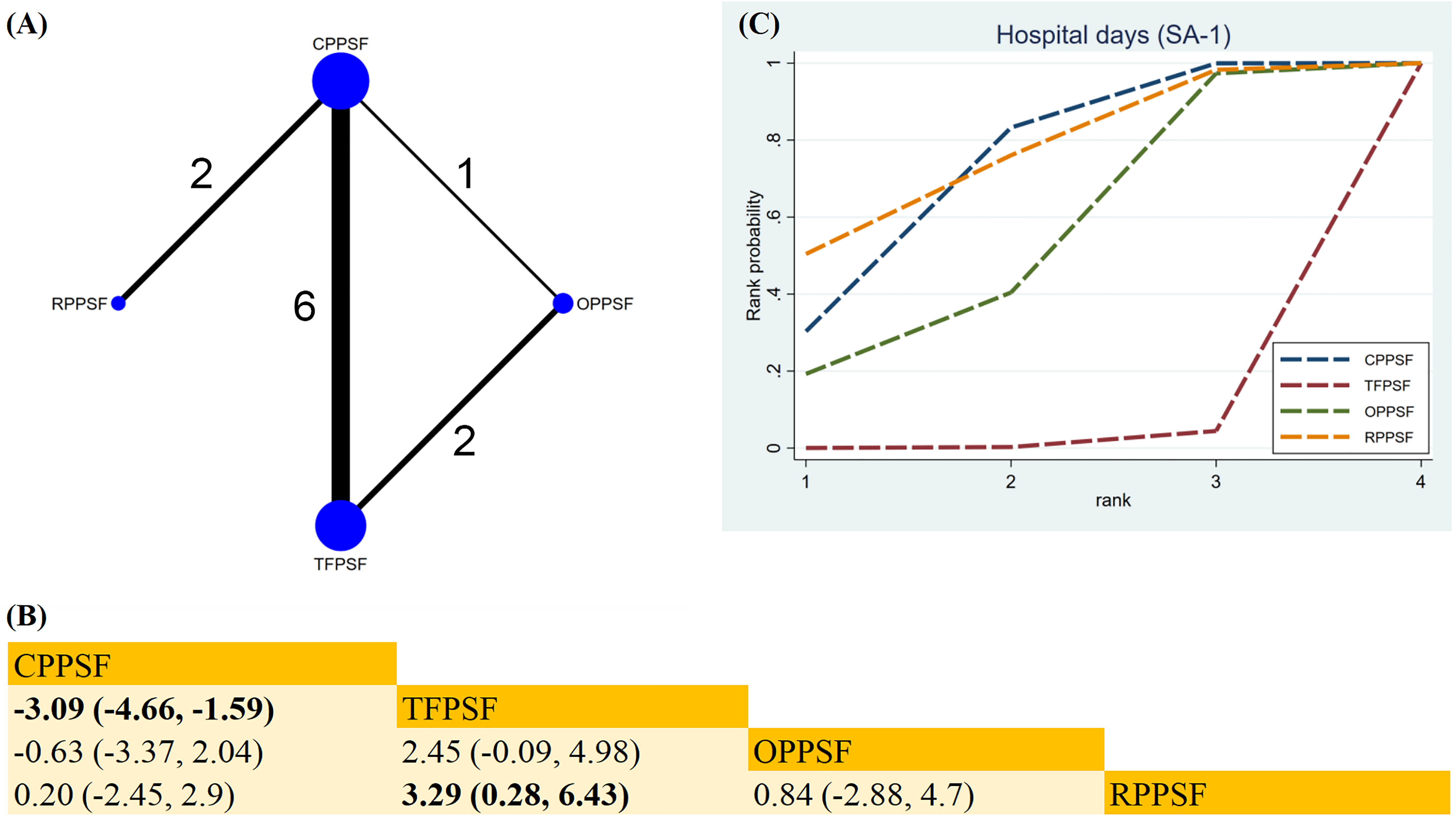

Supplement: Supplementary file 11 — Figure S11: Network plot, SUCRA curve, and comparative outcomes of the dominant country sensitivity analysis. (A) Network plot for hospital days. (B) Relative effects of different surgical approaches on hospital days. (C) SUCRA graph of hospital days. Estimates are shown in MD with 95% CrI. The numbers adjacent to the connecting lines in the network plot indicate the number of studies that directly compared the two corresponding interventions. Comparisons between surgical approaches should be interpreted from left to right. Statistically significant results are highlighted in bold. [file OS-17-3302-s013.tif]

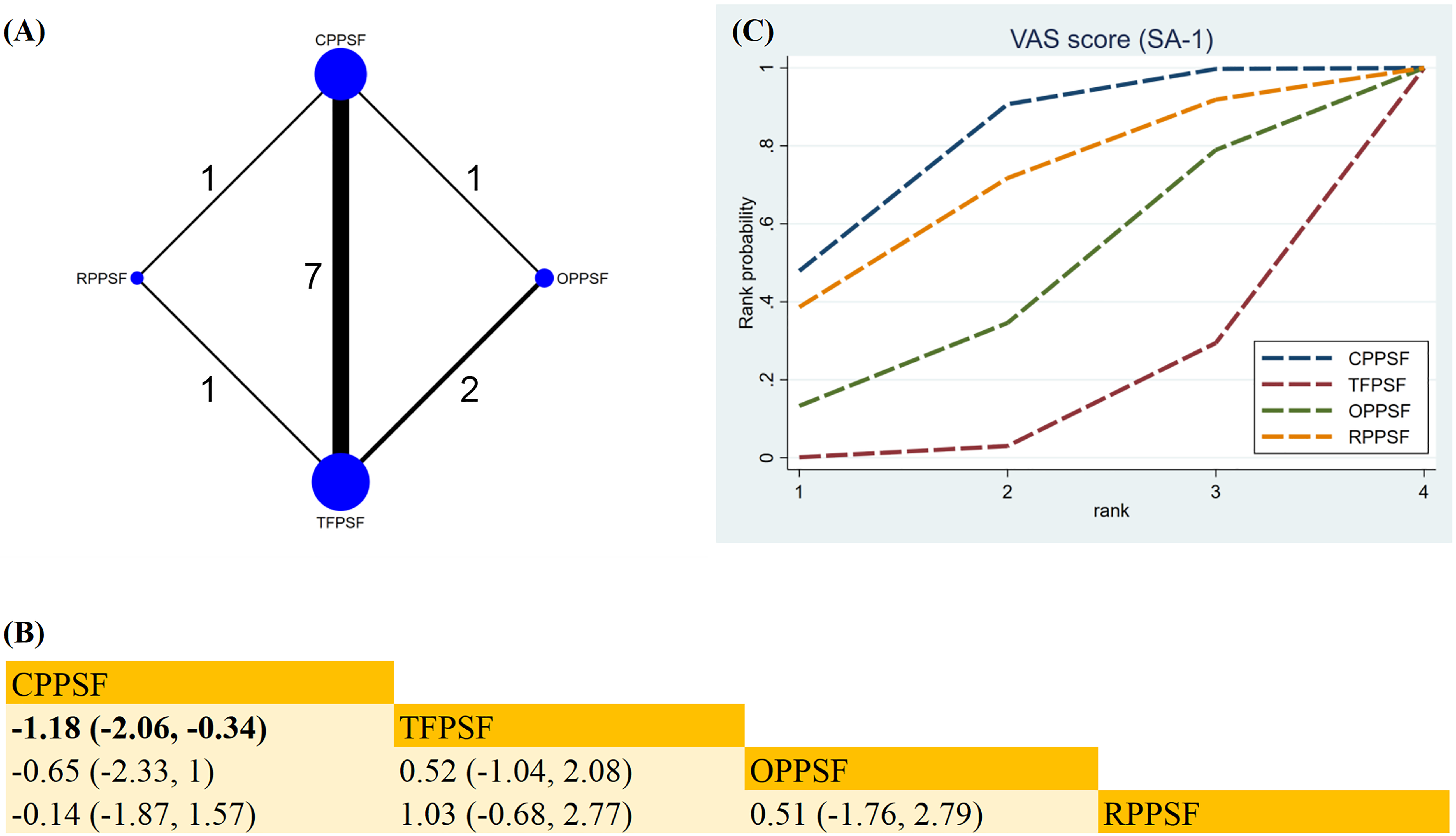

Supplement: Supplementary file 12 — Figure S12: Network plot, SUCRA curve, and comparative outcomes of the dominant country sensitivity analysis. (A) Network plot for VAS score. (B) Relative effects of different surgical approaches on VAS score. (C) SUCRA graph of VAS score. Estimates are shown in MD with 95% CrI. The numbers adjacent to the connecting lines in the network plot indicate the number of studies that directly compared the two corresponding interventions. Comparisons between surgical approaches should be interpreted from left to right. Statistically significant results are highlighted in bold. [file OS-17-3302-s016.tif]

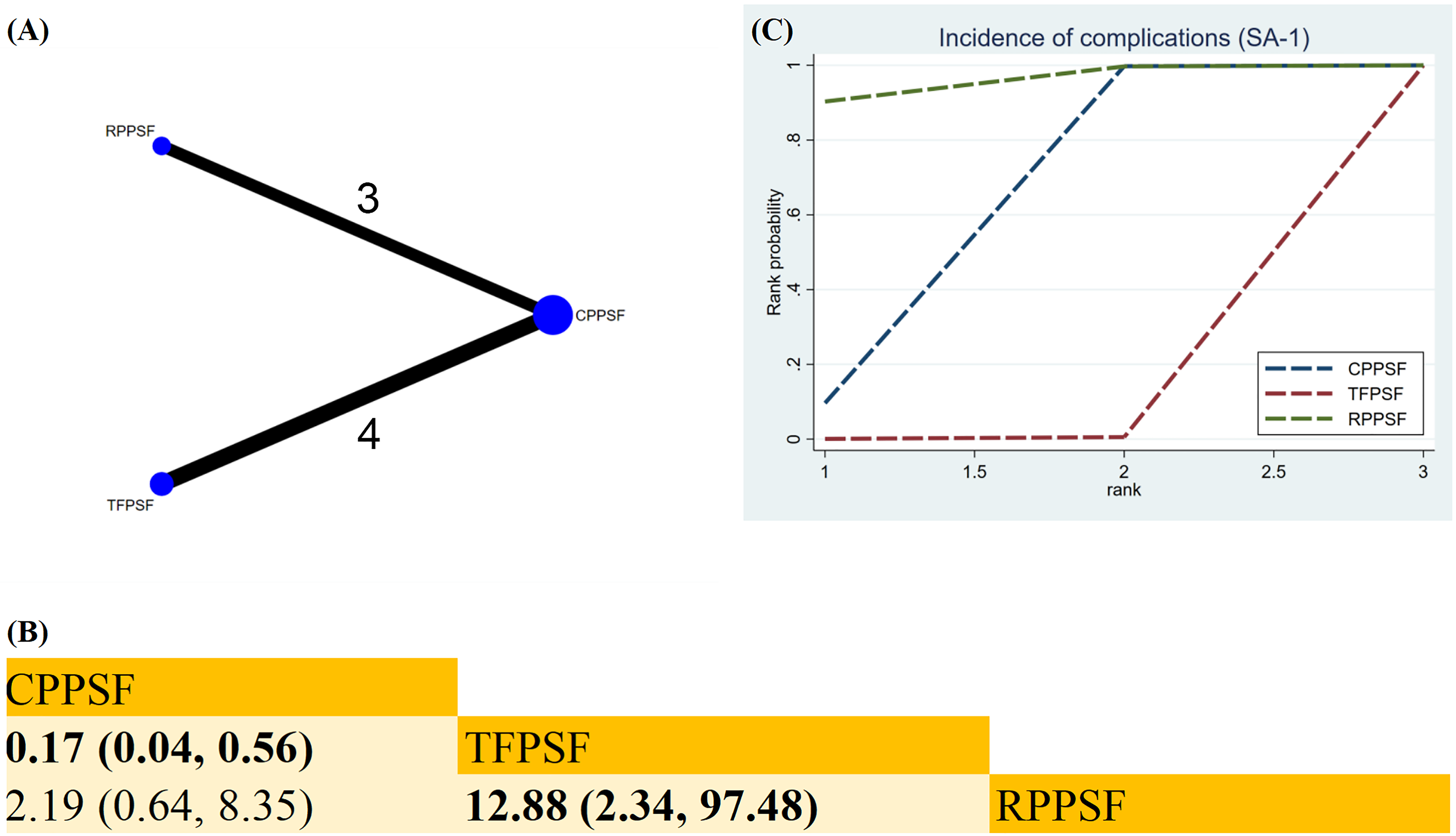

Supplement: Supplementary file 13 — Figure S13: Network plot, SUCRA curve, and comparative outcomes of the dominant country sensitivity analysis. (A) Network plot for incidence of complications. (B) Relative effects of various surgical approaches on the incidence of complications. (C) SUCRA graph of the incidence of complications. Estimates are expressed as RR with 95% CrI. The numbers adjacent to the connecting lines in the network plot indicate the number of studies that directly compared the two corresponding interventions. Comparisons between surgical approaches should be read from left to right. Statistically significant results are highlighted in bold. [file OS-17-3302-s001.tif]

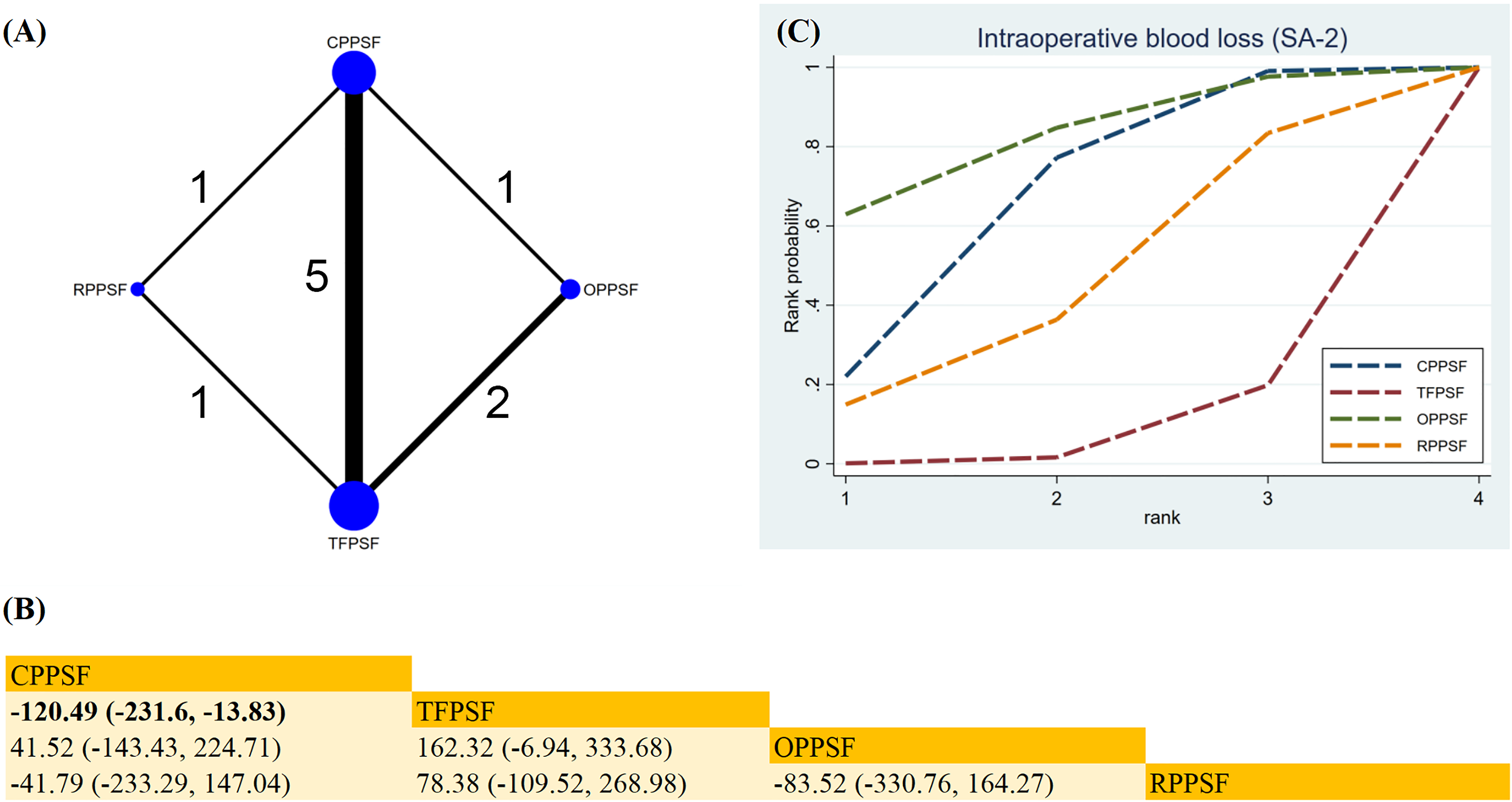

Supplement: Supplementary file 14 — Figure S14: Network plot, SUCRA curve, and comparative outcomes of the high‐quality study sensitivity analysis. (A) Network plot for IBL. (B) Relative effects of different surgical approaches on IBL. (C) SUCRA graph of IBL. Estimates are expressed as MD with 95% CrI. The numbers adjacent to the connecting lines in the network plot indicate the number of studies that directly compared the two corresponding interventions. Comparisons between surgical approaches should be read from left to right. Statistically significant results are highlighted in bold. [file OS-17-3302-s018.tif]

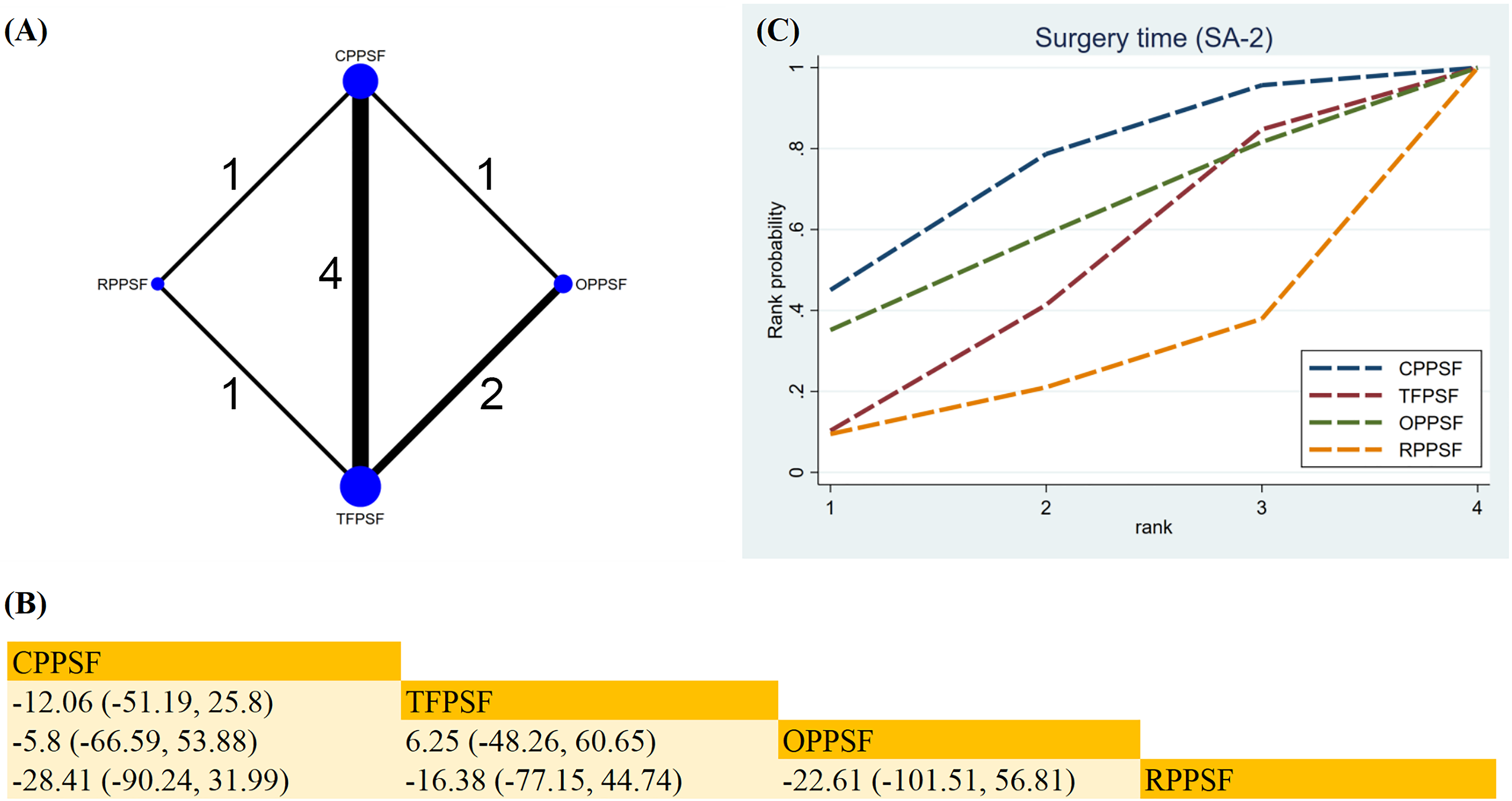

Supplement: Supplementary file 15 — Figure S15: Network plot, SUCRA curve, and comparative outcomes of the high‐quality study sensitivity analysis. (A) Network plot for surgery time. (B) Relative effects of different surgical approaches on surgery time. (C) SUCRA graph of surgery time. Estimates are shown in MD with 95% CrI. The numbers adjacent to the connecting lines in the network plot indicate the number of studies that directly compared the two corresponding interventions. Comparisons between surgical approaches should be interpreted from left to right. Statistically significant results are highlighted in bold. [file OS-17-3302-s008.tif]

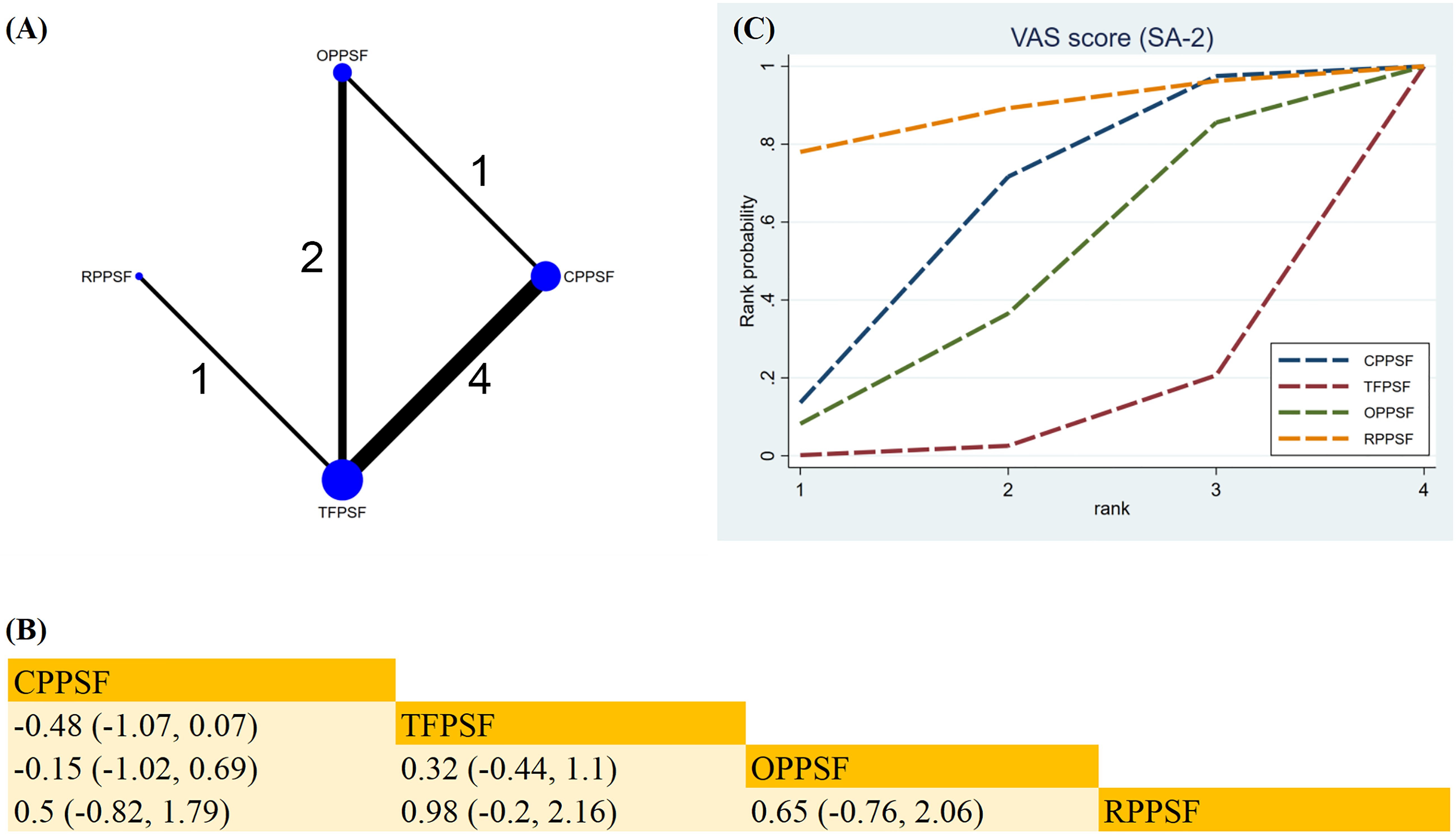

Supplement: Supplementary file 16 — Figure S16: Network plot, SUCRA curve, and comparative outcomes of the high‐quality study sensitivity analysis. (A) Network plot for VAS score. (B) Relative effects of different surgical approaches on VAS score. (C) SUCRA graph of VAS score. Estimates are shown in MD with 95% CrI. The numbers adjacent to the connecting lines in the network plot indicate the number of studies that directly compared the two corresponding interventions. Comparisons between surgical approaches should be interpreted from left to right. Statistically significant results are highlighted in bold. [file OS-17-3302-s007.tif]
