# Supplementary material for: Robot‐Assisted, Conventional Fluoroscopy (C‐Arm), O‐Arm Navigation, and Freehand Pedicle Screw Fixation in Thoracolumbar Spine Fracture Surgery: A Network Meta‐Analysis
Source: Orthop Surg. 2025 Oct 11;17(12):3302–17. doi: 10.1111/os.70189 (PMC12685484; doi:10.1111/os.70189)
Supplement: Supplementary file 17 — Data S1: Retrieval strategy. [file OS-17-3302-s009.docx]

**Supplement material 1 Retrieval strategy**

**Pubmed: 71 records**

#1:"Pedicle Screws"[Mesh]

#2:"Fluoroscopy"[Mesh]

#3:"Robotic Surgical Procedures"[Mesh]

#4:((((((thoracolumbar fracture*[Title/Abstract]) OR (thoracolumbar compression fracture*[Title/Abstract])) OR (thoracolumbar burst fracture*[Title/Abstract])) OR (thoracolumbar spinal fracture*[Title/Abstract])) OR (thoracolumbar spine fracture*[Title/Abstract])) OR (thoracic fracture[Title/Abstract])) OR (lumbar fracture[Title/Abstract])

#5:((Pedicle Screw*[Title/Abstract]) OR (Everest MI[Title/Abstract])) OR (OsseoScrew[Title/Abstract])

#6:freehand[Title/Abstract]

#7:((((((((Fluoroscopy[Title/Abstract]) OR (fluoroscop*[Title/Abstract])) OR (fluorescence*[Title/Abstract])) OR (fluorescent scanning[Title/Abstract])) OR (fluorophotography[Title/Abstract])) OR (photofluoroscopy[Title/Abstract])) OR (radiofluoroscopy[Title/Abstract])) OR (Radiography-guided[Title/Abstract])) OR (x-ray-guided[Title/Abstract])

#8:(((((((((((Robotic Surgical Procedure*[Title/Abstract]) OR (Robotic Assisted Surger*[Title/Abstract])) OR (Robot Assisted Surger*[Title/Abstract])) OR (Robot Surger*[Title/Abstract])) OR (Robot-Enhanced Procedure*[Title/Abstract])) OR (Robot Enhanced Surger*[Title/Abstract])) OR (robot aided surger*[Title/Abstract])) OR (robotic aided surger*[Title/Abstract])) OR (robotic surger*[Title/Abstract])) OR (robotically assisted surger*[Title/Abstract])) OR (robot-assisted[Title/Abstract])) OR (robot-guided[Title/Abstract])

#9:O-Arm[Title/Abstract]

#10:#1 OR #5

#11:#2 OR #3 OR #6 OR #7 OR #8 #9

#12:#4 AND #10 AND #11

**Embase: 146 records**

#1：'thoracolumbar fracture'/exp

#2：'pedicle screw'/exp

#3：'fluoroscopy'/exp

#4: 'robot assisted surgery'/exp

#5:'thoracolumbar fracture*':ab,ti OR 'thoracolumbar compression fracture*':ab,ti OR 'thoracolumbar burst fracture*':ab,ti OR 'thoracolumbar spinal fracture*':ab,ti OR 'thoracolumbar spine fracture*':ab,ti OR 'thoracic fracture':ab,ti OR 'lumbar fracture':ab,ti

#6:'pedicle screw':ab,ti OR 'pedicle screws':ab,ti OR 'everest mi':ab,ti OR osseoscrew:ab,ti

#7:freehand:ab,ti

#8:fluoroscopy:ab,ti OR fluoroscop*:ab,ti OR fluorescence*:ab,ti OR 'fluorescent scanning':ab,ti OR fluorophotography:ab,ti OR photofluoroscopy:ab,ti OR radiofluoroscopy:ab,ti OR 'radiography guided':ab,ti OR 'x ray guided':ab,ti

#9:'robot assisted surgery':ab,ti OR 'robotic surgical procedure*':ab,ti OR 'robotic assisted surger*':ab,ti OR 'robot assisted surger*':ab,ti OR 'robot surger*':ab,ti OR 'robot-enhanced procedure*':ab,ti OR 'robot enhanced surger*':ab,ti OR 'robot aided surger*':ab,ti OR 'robotic aided surger*':ab,ti OR 'robotic surger*':ab,ti OR 'robotically assisted surger*':ab,ti OR 'robot assisted':ab,ti OR 'robot guided':ab,ti
#10:'o arm':ab,ti

#11:#1 OR #5

#12:#2 OR #6

#13:#3 OR #4 OR #7 OR #8 OR #9 OR #10

#14:#11 AND #12 AND #13

**Cochrane Library: 42 records**

#1 MeSH descriptor: [Pedicle Screws] explode all trees

#2 MeSH descriptor: [Fluoroscopy] explode all trees

#3 MeSH descriptor: [Robotic Surgical Procedures] explode all trees

#4 (thoracolumbar fracture*):ti,ab,kw OR (thoracolumbar compression fracture*):ti,ab,kw OR (thoracolumbar burst fracture*):ti,ab,kw OR (thoracolumbar spinal fracture*):ti,ab,kw OR (thoracolumbar spine fracture*):ti,ab,kw (Word variations have been searched)

#5 (thoracic fracture):ti,ab,kw OR (lumbar fracture):ti,ab,kw (Word variations have been searched)

#6 (Pedicle Screw*):ti,ab,kw OR (Everest MI):ti,ab,kw OR (OsseoScrew):ti,ab,kw (Word variations have been searched)

#7 (freehand):ti,ab,kw (Word variations have been searched)

#8 (Fluoroscopy):ti,ab,kw OR (fluoroscop*):ti,ab,kw OR (fluorescence*):ti,ab,kw OR (fluorescent scanning):ti,ab,kw OR (fluorophotography):ti,ab,kw (Word variations have been searched)

#9 (photofluoroscopy):ti,ab,kw OR (radiofluoroscopy):ti,ab,kw OR (Radiography-guided):ti,ab,kw OR (x-ray-guided):ti,ab,kw (Word variations have been searched)

#10 (Robotic Surgical Procedure*):ti,ab,kw OR (Robotic Assisted Surger*):ti,ab,kw OR (Robot Assisted Surger*):ti,ab,kw OR (Robot Surger*):ti,ab,kw OR (Robot-Enhanced Procedure*):ti,ab,kw (Word variations have been searched)

#11 (Robot Enhanced Surger*):ti,ab,kw OR (robot aided surger*):ti,ab,kw OR (robotic aided surger*):ti,ab,kw OR (robotic surger*):ti,ab,kw OR (robotically assisted surger*):ti,ab,kw (Word variations have been searched)

#12 (robot-assisted):ti,ab,kw OR (robot-guided):ti,ab,kw (Word variations have been searched)

#13 (O-Arm):ti,ab,kw (Word variations have been searched)

#14 #1 OR #6

#15 #2 OR #3 OR #7 OR #8 OR #9 OR #10 OR #11 OR #12 OR #13

#16 #4 OR #5

#17 #14 AND #15 AND #16

**Web of Science: 357 records**

#1:"((((((TS=(thoracolumbar fracture*)) OR TS=(thoracolumbar compression fracture*)) OR TS=(thoracolumbar burst fracture*)) OR TS=(thoracolumbar spinal fracture*)) OR TS=(thoracolumbar spine fracture*)) OR TS=(thoracic fracture)) OR TS=(lumbar fracture) and Preprint Citation Index (Exclude–Database)"

#2: "((TS=(Pedicle Screw*)) OR TS=(Everest MI)) OR TS=(OsseoScrew) and Preprint Citation Index (Exclude–Database)"

#3: "TS=(freehand) and Preprint Citation Index (Exclude–Database)"

#4: "((((((((TS=(Fluoroscopy)) OR TS=(fluoroscop*)) OR TS=(fluorescence*)) OR TS=(fluorescent scanning )) OR TS=(fluorophotography)) OR TS=(photofluoroscopy)) OR TS=(radiofluoroscopy)) OR TS=(Radiography-guided)) OR TS=(x-ray-guided) and Preprint Citation Index (Exclude–Database)"

#5:"((((((((((((TS=(robot assisted surgery)) OR TS=(Robotic Surgical Procedure*)) OR TS=(Robotic Assisted Surger*)) OR TS=(Robot Assisted Surger*)) OR TS=(Robot Surger*)) OR TS=(Robot-Enhanced Procedure*)) OR TS=(Robot Enhanced Surger*)) OR TS=(robot aided surger*)) OR TS=(robotic aided surger*)) OR TS=(robotic surger*)) OR TS=(robotically assisted surger*)) OR TS=(robot-assisted)) OR TS=(robot-guided) and Preprint Citation Index (Exclude–Database)"

#6:"TS=(O-Arm ) and Preprint Citation Index (Exclude–Database)"
#7:"#3 OR #4 OR #5 OR #6 and Preprint Citation Index (Exclude–Database)"

#8:"#1 AND #2 AND #7 and Preprint Citation Index (Exclude–Database)"
