# Supplementary material for: Robot‐Assisted, Conventional Fluoroscopy (C‐Arm), O‐Arm Navigation, and Freehand Pedicle Screw Fixation in Thoracolumbar Spine Fracture Surgery: A Network Meta‐Analysis
Source: Orthop Surg. 2025 Oct 11;17(12):3302–17. doi: 10.1111/os.70189 (PMC12685484; doi:10.1111/os.70189)
Supplement: Supplementary file 19 — Table S1: SUCRA values of four surgical techniques according to outcomes. [file OS-17-3302-s012.docx]

Table S1 SUCRA values of four surgical techniques according to outcomes

|  | Accuracy rate of pedicle screw placement | Intraoperative blood loss | Surgery time | Hospital days | VAS score | Cobb angle | Incidence of complications |
| --- | --- | --- | --- | --- | --- | --- | --- |
| TFPSF | 16.4% | 1.2% | 21.1% | 9.3% | 10.3% | 47.4% | 0.2% |
| CPPSF | 18.2% | 67.6% | 81.6% | 64.7% | 77.9% | 72.4% | 55.0% |
| OPPSF | 92.7% | 79.8% | 44.3% | 61.0% | 42.8% | NA | NA |
| RPPSF | 72.7% | 51.4% | 52.9% | 65.0% | 69.0% | 30.2% | 94.9% |
