# Supplementary material for: Robot‐Assisted, Conventional Fluoroscopy (C‐Arm), O‐Arm Navigation, and Freehand Pedicle Screw Fixation in Thoracolumbar Spine Fracture Surgery: A Network Meta‐Analysis
Source: Orthop Surg. 2025 Oct 11;17(12):3302–17. doi: 10.1111/os.70189 (PMC12685484; doi:10.1111/os.70189)
Supplement: Supplementary file 20 — Table S2: Results of local inconsistency analysis. [file OS-17-3302-s015.docx]

Table S2 Results of local inconsistency analysis

| Outcomes | Comparison | Direct | Indirect | Network | P.value CrI |
| --- | --- | --- | --- | --- | --- |
| Accuracy rate of pedicle screw placement | CPPSF vs TFPSF | 0.67 (-0.71, 2.2) | -0.88 (-2.6, 0.86) | 0.058 (-0.93, 1.1) | 0.130475 |
|  | CPPSF vs OPPSF | 1.0 (-0.00054, 2.1) | 3.2 (0.85, 5.7) | 1.4 (0.43, 2.4) | 0.090675 |
|  | CPPSF vs RPPSF | 1.2 (0.41, 2.1) | 1.0 (-1.4, 3.3) | 1.2 (0.40, 2.0) | 0.825975 |
|  | TFPSF vs OPPSF | 1.9 (0.43, 3.3) | 0.28 (-1.8, 2.3) | 1.3 (0.22, 2.5) | 0.17285 |
|  | TFPSF vs RPPSF | 0.94 (-0.59, 2.4) | 1.6 (-0.10, 3.5) | 1.1 (0.048, 2.2) | 0.499475 |
| Intraoperative blood loss | CPPSF vs TFPSF | 1.4e+02 (75., 2.1e+02) | 25. (-2.1e+02, 2.6e+02) | 1.4e+02 (73., 2.1e+02) | 0.294725 |
|  | CPPSF vs OPPSF | -2.7 (-2.1e+02, 2.0e+02) | -1.1e+02 (-3.4e+02, 1.1e+02) | -28. (-1.7e+02, 1.2e+02) | 0.4199 |
|  | CPPSF vs RPPSF | -4.4 (-1.2e+02, 1.1e+02) | 1.2e+02 ( -98., 3.4e+02) | 24. ( -81., 1.3e+02) | 0.291625 |
|  | TFPSF vs RPPSF | -29. (-2.4e+02, 1.8e+02) | -1.5e+02 (-2.9e+02, -17.) | -1.1e+02 (-2.3e+02, -1.7) | 0.290175 |
| Surgery time | CPPSF vs TFPSF | 26. (5.4, 48.) | -24. (-81., 34.) | 21. (-0.52, 42.) | 0.0979 |
|  | CPPSF vs OPPSF | -4.5 (-53., 44.) | 39. (-33., 1.1e+02) | 13. ( -26., 52.) | 0.29645 |
|  | CPPSF vs RPPSF | -5.7 ( -41., 29.) | 60. (-4.1, 1.3e+02) | 9.3 ( -25., 44.) | 0.0749 |
|  | TFPSF vs OPPSF | 4.1 ( -43., 51.) | -8.9 (-80., 63.) | -7.4 (-46., 32.) | 0.747225 |
|  | TFPSF vs RPPSF | 35. ( -27., 97.) | -31. ( -72., 10.) | -11. ( -49., 26.) | 0.0747 |
| Hospital days | CPPSF vs OPPSF | -0.20 (-7., 6.6) | 0.31 (-7.1, 7.6) | 0.041 (-4.2, 4.3) | 0.9127 |
| VAS score | CPPSF vs TFPSF | 1.0 (0.23, 1.9) | 1.1 (-2.4, 4.5) | 1.0 (0.38, 1.7) | 0.976275 |
|  | CPPSF vs OPPSF | 0.0031 (-2.3, 2.3) | 0.84 (-1.6, 3.3) | 0.54 (-0.87, 2.) | 0.573625 |
|  | CPPSF vs RPPSF | 0.11 (-2.1, 2.3) | 0.042 (-2.3, 2.4) | 0.074 (-1.4, 1.6) | 0.961775 |
|  | TFPSF vs RPPSF | -0.98 (-3.2, 1.3) | -0.92 (-3.3, 1.4) | -0.95 (-2.4, 0.54) | 0.970725 |
