# Supplementary material for: Robot‐Assisted, Conventional Fluoroscopy (C‐Arm), O‐Arm Navigation, and Freehand Pedicle Screw Fixation in Thoracolumbar Spine Fracture Surgery: A Network Meta‐Analysis
Source: Orthop Surg. 2025 Oct 11;17(12):3302–17. doi: 10.1111/os.70189 (PMC12685484; doi:10.1111/os.70189)
Supplement: Supplementary file 21 — Table S3: SUCRA values of four surgical techniques according to outcomes in the dominant country sensitivity analysis. [file OS-17-3302-s021.docx]

Table S3 SUCRA values of four surgical techniques according to outcomes in the dominant country sensitivity analysis

|  | Accuracy rate of pedicle screw placement | Intraoperative blood loss | Surgery time | Hospital days | VAS score | Incidence of complications |
| --- | --- | --- | --- | --- | --- | --- |
| TFPSF | 17.6% | 2.4% | 30.3% | 1.6% | 10.9% | 0.3% |
| CPPSF | 17.1% | 60.2% | 75.5% | 71.2% | 79.4% | 54.7% |
| OPPSF | 90.6% | 87.8% | 45.0% | 52.3% | 42.3% | NA |
| RPPSF | 74.6% | 49.6% | 49.2% | 74.9% | 67.4% | 95.0% |
