# Supplementary material for: Robot‐Assisted, Conventional Fluoroscopy (C‐Arm), O‐Arm Navigation, and Freehand Pedicle Screw Fixation in Thoracolumbar Spine Fracture Surgery: A Network Meta‐Analysis
Source: Orthop Surg. 2025 Oct 11;17(12):3302–17. doi: 10.1111/os.70189 (PMC12685484; doi:10.1111/os.70189)
Supplement: Supplementary file 22 — Table S4: SUCRA values of four surgical techniques according to outcomes in the high‐quality study sensitivity analysis. [file OS-17-3302-s014.docx]

Table S4 SUCRA values of four surgical techniques according to outcomes in the high-quality study sensitivity analysis

|  | Intraoperative blood loss | Surgery time | VAS score |
| --- | --- | --- | --- |
| TFPSF | 7.2% | 45.5% | 7.8% |
| CPPSF | 66.1% | 73.1% | 60.9% |
| OPPSF | 81.8% | 58.6% | 43.4% |
| RPPSF | 44.9% | 22.8% | 87.8% |
